# Selecting indicators for the measurement of low-value care using German claims data: A three-round modified Delphi panel

**DOI:** 10.1371/journal.pone.0314864

**Published:** 2025-02-18

**Authors:** Carolina Pioch, Anne Neubert, Lotte Dammertz, Hanna Ermann, Meik Hildebrandt, Peter Ihle, Monika Nothacker, Udo Schneider, Enno Swart, Reinhard Busse, Verena Vogt

**Affiliations:** 1 Department of Health Care Management, Technical University of Berlin, Berlin, Germany; 2 Department of Orthopaedics and Traumatology, Medical Faculty and University Hospital Düsseldorf, Heinrich-Heine-University Düsseldorf, Düsseldorf, Germany; 3 Department of Epidemiology and Health Care Atlas, Central Research Institute for Ambulatory Health Care in Germany, Berlin, Germany; 4 PMV Research Group at the Department of Psychiatry and Psychotherapy for Children and Young Adults, University Hospital Cologne, Cologne, Germany; 5 Institute for Medical Knowledge Management, Association of the Scientific Medical Societies in Germany, Berlin, Germany; 6 Techniker Krankenkasse, Hamburg, Germany; 7 Institute of Social Medicine and Health Systems Research, Otto von Guericke University Magdeburg, Magdeburg, Germany; 8 Institute of General Practice and Family Medicine, Jena University Hospital, Jena, Germany; IST: Universidade de Lisboa Instituto Superior Tecnico, PORTUGAL

## Abstract

By reducing healthcare services that offer little benefit or potential harm to patients (low-value care), resources can be redirected towards more adequate treatments, improving healthcare efficiency and patient outcomes. This study aimed to systematically incorporate clinical expertise across medical disciplines through a Delphi process to establish indicators for measuring low-value care, ensuring their acceptance by medical societies, the broader medical community, and patients. We developed two versions (one with higher sensitivity and one with higher specificity) for almost each of the 42 indicators identified as potentially measurable in a previous systematic review. We conducted a three-round modified Delphi panel based on the RAND/UCLA appropriateness methodology, with 62 experts from 52 Scientific Medical Societies and professional organisations, and patient representatives. In round one, each indicator was rated for its ability to indicate low-value healthcare and its measurability in German claims data. This was followed by an online discussion in round two. The indicators were then modified based on expert feedback and re-assessed in round three. As a result, 24 indicators were deemed appropriate for measuring low-value care, covering areas such as pharmaceuticals, diagnostic tests, screening, and treatment. For example, one indicator identified patients with cancer who received chemotherapy in the last month of life. These indicators will help identify healthcare services that may require policy-level interventions to improve the quality of care. However, most low-value care indicators can only be measured in German claims data if documentation requirements for relevant information are expanded.

## Introduction

Avoiding overuse and ensuring that resources are allocated to healthcare services that provide clinical benefits to patients is becoming more crucial in light of the growing shortage of healthcare professionals and rising healthcare costs [1]. By reducing healthcare services that offer very little or no benefit to patients (low-value care), resources can be redirected towards more high-value care, potentially improving the efficiency of the healthcare system and patient outcomes.

Several international initiatives have been launched to reduce the provision of low-value. For example, the Choosing Wisely® campaign encourages participating medical societies to develop recommendations that identify routinely performed yet medically unnecessary healthcare services which may also harm patients [2]. Some studies translate these recommendations into indicators that directly measure the prevalence of low-value care using claims data [3, 4]. Claims data provide information on population level, are widely accessible, comprehensive in terms of services relevant for billing purposes, and unbiased regarding services covered by statutory health insurance [5, 6]. These indicators could be used by policymakers, insurers, and researchers to measure the prevalence of low-value care to help distinguish between low-value and appropriate care [5] and identify areas where the delivery of healthcare services seems to differ from published recommendations [4, 7].

So far, the measurement of low-value care has been documented across various healthcare systems, especially in high-income countries, like the U.S., Canada, and Australia [8]. For example, about 41% of all Medicare beneficiaries in the U.S. receive at least one of 26 identified low-value healthcare services, accounting for 2.7% of annual healthcare spending [4]. In Australia, up to 19.2% of 27 selected low-value healthcare services are associated with 14.7% of the total inpatient costs for all episodes involving these services [7]. In the German healthcare system, efforts to measure low-value care primarily revolved around single aspects of healthcare, such as radiological imaging for back pain [9] or end-of-life care [10, 11]. There is currently no comprehensive overview of how to directly measure overuse in the German healthcare system and the feasibility of using claims data for measuring low-value care remains largely unexplored. This may be due to specific coding practices in German claims data. For example, outpatient doctor-patient-contacts are partly billed on a per capita payment basis, usually during the first visit in a quarter [12]. Consequently, diagnoses are not available on a daily basis, making it difficult to establish a temporal link between diagnoses and potential low-value healthcare services, such as diagnostic procedures or therapies. Additionally, German claims data lack clinical information on disease severity, disease progression, or laboratory results, which could complicate decisions regarding the appropriateness of specific healthcare services.

In this study, we aim to assess which of the previously published low-value care indicators can be used to measure the prevalence of low-value care in the German healthcare system. We apply the Delphi method with representatives of German Scientific Medical Societies and patient organisations to systematically incorporate clinical expertise across a wide range of medical disciplines to establish indicators for measuring low-value care, ensuring their acceptance by medical societies, the broader medical community, and patients. We carry this study out as part of the research project “IndiQ” (development of a tool for measuring indication quality in claims data and identification of needs and strategies for action). The selected indicators will be used to quantify the number of low-value care services billed by physicians in the statutory health insurance system, helping to identify potential healthcare services and areas for improvement in the quality of care.

## Methods

The reporting of the conducted Delphi panel adheres to the reporting guidelines for Delphi techniques in health sciences as outlined by Spranger et al. [13] as they offer a more comprehensive framework than previous guidelines such as those by Boulkedid et al. [14]. While Boulkedid et al. focus on three core aspects (Questionnaires, Experts, and Rounds) [14], Spranger et al. propose a broader, nine-category framework, encompassing areas like Expert Panel, Survey Design, Process Regulation, and Ethics [13], which aligns with our objective of providing a detailed description of the Delphi process.

### List of potential indicators

We adopted a methodological framework suggested by Chalmers et al. and defined the indicators as follows:

- Numerator: The number of patients who received the potential low-value healthcare service
- Denominator (patient-based): All patients who were eligible for the low-value healthcare service
- Denominator (service-based): All cases where the healthcare service was provided [15].

[15] In a literature review described elsewhere, we identified 171 indicators used in international literature to detect potential low-value healthcare services. After pre-assessing the measurability of these indicators in German claims data and their relevance to the German context, we included 42 indicators in this study [PROSPERO CRD42021235336, publication currently in progress]. We translated the indicators from international literature to ensure compatibility with German claims data by aligning them with the International Classification of Diseases 10^th^ revision German Modification (ICD-10-GM) codes, Operation and Procedure Classification System (OPS) codes, the uniform value scale (“Einheitlicher Bewertungsmaßstab” (EBM)) codes, the Anatomical Therapeutic Chemical (ATC) codes, timing of care, site of care, and demographic information. When codes were not directly transferable, for example, due to ambiguous descriptions of procedures, we consulted with medical experts from our project team’s network who had the relevant expertise.

For almost every indicator, we developed two versions: one with higher sensitivity (and lower specificity) and the other with higher specificity (and lower sensitivity) for detecting low-value healthcare services, as proposed by Schwartz et al. [4]. For example, chemotherapy in the last three months of life for patients with a cancer diagnosis is more sensitive (more low-value healthcare services are detected), whereas chemotherapy in the last month of life for these patients is more specific (smaller proportion of appropriate healthcare services is misclassified as low-value care).

### Critical appraisal and structured consensus with RAND/UCLA method

We used a modified version of the Delphi method based on the Research ANd Development (RAND)/University of California, Los Angeles (UCLA) appropriateness methodology. Between two rounds of questionnaires, experts have the opportunity to discuss their ratings with one another [16], which facilitates clarification while surveying a geographically scattered group [17]. Each expert provides their ratings individually, without interaction during the rating process. After each round of questionnaires, the opinions of all experts are gathered anonymously and circulated back to them [18].

### Composition of the Delphi panel

We assigned each operationalised indicator to the relevant medical disciplines involved in the diagnosis, treatment or prevention of the specified condition and healthcare service described by the indicator. Then, we matched these medical disciplines with their respective German Scientific Medical Societies (“Fachgesellschaften”) or professional organisations. With the support of the Association of the Scientific Medical Societies in Germany (“Arbeitsgemeinschaft der Wissenschaftlichen Medizinischen Fachgesellschaften e.V.” (AWMF)), we sent invitation letters via e-mail to these societies, requesting the appointment of a representative and a substitute to participate in the panel. The letters provided general information about the indicators and an overview of the IndiQ research project. Over four months, we followed up with two e-mail reminders and a phone call if necessary. If we did not receive a response, we contacted the German Association of Statutory Health Insurance Physicians ("Kassenärztliche Bundesvereinigung") or the respective professional organisation to nominate a representative. We recruited all participants from September 7, 2021, to January 10, 2022.

In line with the RAND/UCLA methodology, we aimed to have at least seven representatives rating each indicator, representing the respective clinical area to ensure sufficient diversity [16]. However, we were not able to achieve this goal for every indicator. Some indicators involved only a few or even a single clinical area in the diagnosis, treatment, and prevention of the condition (for example, retinal laser therapy or cryotherapy for asymptomatic lattice degeneration). S1 Table provides an overview of the contacted Scientific Medical Societies and professional organisations.

Furthermore, we requested patient representatives from the G-BA for 16 indicators where no evidence-based guidelines were available. For the remaining 26 indicators, we did not invite patient representatives as they were already involved in the development of evidence-based guidelines in Germany that informed the respective indicator.

All participating representatives and their substitutes provided written consent to participate in the Delphi panel prior to the first survey. We also asked to disclose any potential conflicts of interest that could affect their professional judgments during the panel. After completing the first survey round, we informed the representatives about a financial compensation they would receive upon the completion of all survey rounds. The representatives received a lump sum of 100 Euros for their participation in the Delphi panel and 45 Euros for each indicator they rated. The financial compensation was funded by the project. Ethical approval was not needed, as this study involved participants providing professional opinions and expertise rather than patients or study subjects.

### Delphi process according to RAND/UCLA

The Delphi process consisted of three rounds. The first and last rounds were conducted as online surveys, while the second round involved virtual panel discussions. We piloted the survey with three participants to ensure a logical sequence of questions and clarity of each item. The participants involved in the pilot study were not among the appointed representatives. Based on their feedback, we made structural amendments to the questionnaire. Along with a link to the survey, the representatives received background information on their assigned indicators, including the operational codes used for the sensitive and specific definitions, the literature from which the indicator was originally extracted, the evidence supporting each indicator, and a classification of the evidence according to the Oxford Level of Evidence. Whenever possible, we provided the corresponding recommendations from the Choosing Wisely® initiative and the AWMF Ad Hoc Commission “Deciding Wisely Together” (“Gemeinsam Klug Entscheiden”) (S1 File).

In the first survey round, we asked the representatives to anonymously review the indicators assigned to their respective medical disciplines and rate each indicator based on the following criteria: 1) whether they believed it captured low-value healthcare services, 2) if they agreed with its operationalisation for use in measuring low-value care in German claims data, and 3) if they found the evidence base to be convincing. To establish a shared understanding of when a healthcare service is not considered of low-value, we provided the following definition: The procedure is appropriate if “the expected health benefit […] exceeds the expected negative consequences […] by a sufficiently wide margin that the procedure is worth doing”. We instructed the representatives to assess the indicators based on their own clinical judgement, considering a typical patient presenting to an average physician who performs the procedure in an average healthcare setting, without taking financial implications into account [16]. To pre-assess if the indicators defined low-value healthcare services, we asked the representatives to only rate the sensitive definition of the indicators (as described above) in the first survey round. All 42 potential indicators were formatted into a survey using the online platform www.limesurvey.org.

In the first round, we presented each indicator along with its sensitive definition followed by a statement that needed to be rated (Table 1). Any limitations regarding its measurability in German claims data were listed if applicable. Each indicator could be commented on using free-text fields. The ratings were made individually, with no interaction among the representatives. Round one allowed six weeks for response. We followed up with non-responders via e-mail and phone.

**Table 1:** Questionnaire for the first Delphi round rating indicators for low-value care (online survey).

Number	Statement/ Question	Answer options
1a.	To what extent do you agree or disagree with the statement described above?	1 (= disagree completely) - 9 (= agree completely), “I cannot make a statement”.^a^
1b.	If you do not agree with the indicator (1–3), please explain.	“Content not correct”, “Service not definitely low-value care”, “Service not definitely low-value care for the defined population”, “Not measurable”, “Other”.
2.	Do you find the evidence base convincing?	“Yes”, “No”, “Uncertain^b^”.
3a.	The service described by the indicator is low-value care for which of the following diagnoses?	ICD-10-GM-codes used for the operational definition of the indicator.
3b.	If no code was chosen, please explain.	“The service described by the indicator is not low-value care for any of the codes listed”, “I have no opinion on the diagnoses”, “Other”.
4a.	Which of the codes can be used to measure the service described by the indicator in claims data?	ATC-codes, EBM-codes and OPS-codes used for the operational definition of the indicator.
4b.	If no code was chosen, please explain.	“None of the codes listed would be low-value care for any of the codes used for the operational definition of the indicator”, “I have no opinion on the services”, “Other”.
5.	To what extent do you agree with the following statement: The indicator can be used to detect potential low-value healthcare services.	1 (= disagree completely) - 9 (= agree completely), “I cannot make a statement”.^a^

Abbreviations: ATC = Anatomical Therapeutic Chemical; EBM = uniform value scale (“Einheitlicher Bewertungsmaßstab”); ICD-10-GM = International Classification of Diseases 10^th^ revision German Modification; OPS = Operation and Procedure Classification System.

Note: Patient representatives scored their assigned indicators on a Likert scale ranging from one (= disagree completely) to nine (= agree completely), presented by questions one and five [16].

^a^: Following the RAND/UCLA approach, only the endpoints of the Likert scale were labelled [16].

^b^: “Uncertain” indicates the expert’s inability to conclude whether the evidence presented was sufficient to determine if the indicator detected potential low-value care.

After the first survey, each representative received an individualised document for each of their assigned indicators, showing the distribution of all the representatives’ scores (frequency of the answer options) and the average scores, along with their own ratings. This allowed each representative to examine their own score in the light of the ratings of the other participating representatives. All free-text comments were included in the document anonymously. An evaluation example is presented in S1 Fig. Prior to round two, we made various changes to the indicators’ formulations and operational definitions based on the feedback received from the representatives in the first survey round.

In the second round, the representatives discussed the ratings of each indicator in a moderated online meeting. The meeting focused on determining whether discrepancies in ratings were due to genuine clinical disputes regarding the procedure’s usage or simply due to misunderstanding, rather than reaching consensus [16]. The online discussions were carefully moderated to ensure balanced participation from all representatives. The moderator actively sought input from all members, and any dominant behaviour was managed to maintain a fair and open discussion environment. We asked representatives who were unable to participate in the meeting to send their comments regarding their rated indicators before the second round, ensuring that their opinions could be considered despite their absence. The sensitive and specific definitions and operationalisations of the indicators were then modified and re-sent to all representatives.

In the third round, we once again asked the representatives to anonymously re-rate whether the sensitive and the specific definition of each assigned indicator depicted low-value healthcare services and their measurability. As in the first round, we conducted this re-rating using LimeSurvey. Round three provided a four-week response period. We reminded non-responders via e-mail and phone.

### Appropriateness rating according to RAND/UCLA

We classified each indicator as “appropriate”, “uncertain” or “inappropriate” for measuring the respective low-value health service based on the final median score on the Likert scale (1–3 was defined as inappropriate, 4–6 as uncertain and 7–9 as appropriate). When indicators fell exactly between the three-point boundaries, which are 3.5 and 6.5, we included these medians in the higher appropriateness category, thereby accepting a bias towards making indicators appropriate. In addition, we further categorised the indicators classified as appropriate into levels of agreement to distinguish indicators rated “with agreement” from those rated “with disagreement”. Indicators were categorised “with agreement” when 80% of the ratings fell within the same range as the observed median (1–3; 4–6; 7–9) and “with disagreement” when 20% or more of the ratings fell outside the same range as the observed median. In this context, “disagreement” referred to a lack of consensus, either because the group was divided in their opinions or because opinions were dispersed throughout the whole range of rating possibilities (1 to 9) [16].

Finally, we selected all indicators rated as appropriate for measuring low-value care in German claims data. Indicators rated as appropriate but “with disagreement” were further revised and discussed in online meetings with the respective representatives to exclude the possibility that the disagreement arose from misunderstandings. We refined these indicators if necessary, leading to their inclusion or exclusion. Indicators rated as inappropriate or uncertain were excluded. We circulated all results to the representatives in the form of a report. The report included the final ratings of each indicator presented in the same format as in the first survey round, along with a flow chart of the selection process, highlighting each indicator’s classification of appropriateness and level of agreement.

## Results

The 42 indicators assessed by the representatives in the Delphi panel encompassed the following service areas: four indicators regarding screening (9.5%), 16 indicators covering treatment (38.1%), 13 indicators addressing diagnostic tests (31.0%) and nine indicators from the pharmaceutical field (21.4%) (S2 Table).

### Composition of the Delphi panel

In total, we contacted 52 German Scientific Medical Societies and professional organisations. They nominated 68 representatives, of whom 62 representatives (91.2%) participated in the Delphi panel, including three registered nurses and a medical advisor. These representatives represented 41 different Scientific German Medical Societies and professional organisations, covering 32 distinct medical specialties (S2 File). About one quarter (24.2%) of the representatives were female. Nearly 70% of the representatives were employed in the inpatient sector, with 37.1% working in university hospitals and 32.3% in other hospitals. A total of 14.5% of the representatives worked in the outpatient sector, such as ambulatory practices. Most representatives were involved in both clinical practice and research (61.3%), while some solely worked as physicians (14.5%) or researchers (6.4%). Among the three nurses (4.8%), two were also engaged in research. Furthermore, eight patient representatives participated (12.9%) in the panel. No conflicts of interest were disclosed by any of the participants.

### Delphi process

In the first round of the panel, 62 representatives completed the survey. Each representative reviewed an average of 2.9 indicators. The sensitive definitions of 32 out of the 42 indicators (76.2%) received a median score of ≥ 7, showing that the representatives agreed with the majority of the selected indicators. One indicator (“retinal laser therapy or cryotherapy for asymptomatic lattice degeneration”) was excluded from further consideration after the first survey round due to being classified as unmeasurable in German claims data.

During the second round, conducted on four separate days, 37 of the 62 representatives (59.7%) discussed the remaining 41 indicators in a moderated online panel. Following these discussions, the two indicators related to mammography screening were also excluded due to their lack of measurability in German claims data. The modified versions of the remaining 39 indicators were then re-rated by the experts.

In the third round, a total of 57 representatives out of the remaining 60 representatives (95.0%) participated, as the indicators assigned to two representatives were no longer part of the study. Four indicators fell exactly between the three-point boundaries, with scores of 3.5 (sensitive definitions of the indicators “cancer screening for dialysis-dependent chronic kidney disease” and “tumour marker testing without cancer diagnosis”) and 6.5 (specific definition of the indicator “colorectal cancer screening for older persons” and the sensitive definition of the indicator “abdominal hysterectomy for benign diseases”). Overall, 27 sensitive definitions and 29 specific definitions obtained a median score of ≥ 6.5 and were classified as appropriate. Only ten of the indicators (25.6%) were reviewed by at least seven representatives.

**Fig 1:**
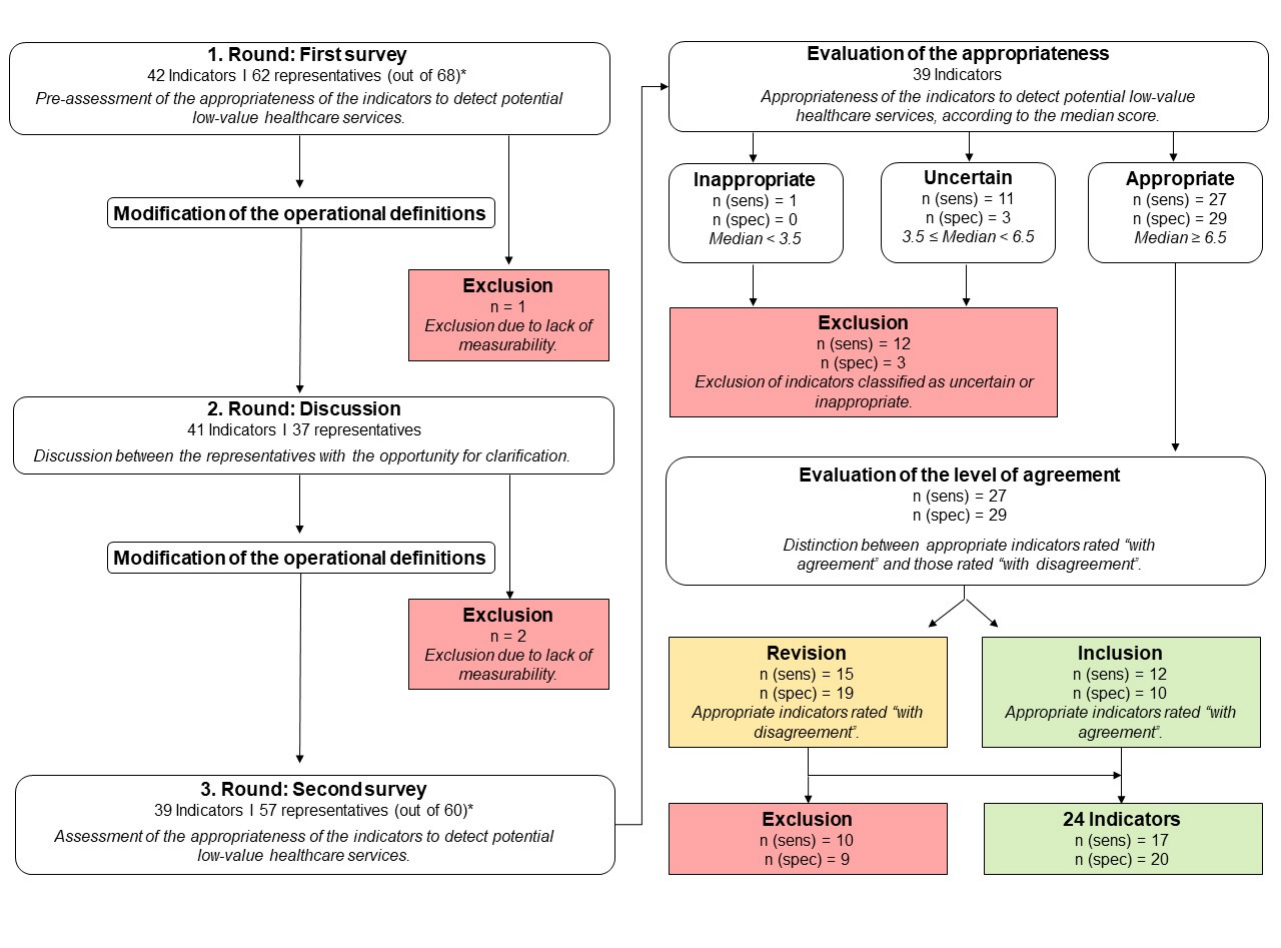
Delphi process of the indicator selection according to RAND/UCLA. Abbreviations: sens = sensitive; spec = specific. *In total, 68 representatives were nominated to participate in the first round of the Delphi panel. Due to the exclusion of three indicators, only 60 out of the initial 62 participating representatives were invited to participate in round three.

Comparing the median ratings of sensitive indicator definitions between round one and round three, 21 indicators received lower ratings (53.8%), eleven remained unchanged (28.2%), and seven received higher ratings (17.9%). In total, twelve of the 27 sensitive definitions (44.4%) and ten (34.5%) of the 29 specific definitions classified as appropriate were rated with agreement. Further revision and refinement of the indicators rated with disagreement resulted in a final selection of 24 indicators, including 17 sensitive and 20 specific definitions, to measure low-value care in German claims data. The final selection included one indicator related to screening, eight covering treatment, eight related to diagnostic tests, and seven from the pharmaceutical field. The decisions from all rounds are shown in Table 2. Fig 1 provides an overview of the entire indicator selection process. The number of respondents per indicator are presented in S3 Table. All data is provided in S1 Dataset.

**Table 2:** Final Delphi ratings of each indicator indicating low-value care.

Indicator	Round 1^a^		Round 3			Classifica- tion A/U/I	Agree-ment^d^ Y/N	Inclusion^e^ Y/N
	Median^b^ (Range)	Validity^c^ Y/N	Appropriate-ness of…	Median^b^ (Range)	Validity^c^ Y/N			
**Pharmaceuticals**								
**Acid blockers for uncomplicated gastroesophageal reflux**	8.5 (1)	N	Sens	5.5 (7)	N	U	-	N
			Spec	8.5 (1)		A	Y	Y
**Antibiotics for acute otitis media**	9 (4)	N	Sens	9 (2)	N	A	Y	Y
			Spec	7 (3)		A	N	Y^f^
**Antibiotics for uncomplicated respiratory tract infections**	9 (2)	Y	Sens	8 (8)	Y	A	N	Y^f^
			Spec	7 (8)		A	Y	Y
**Antipsychotics as first choice for dementia**	7 (7)	N	Sens	4.5 (8)	N	U	-	N
			Spec	4.5 (8)		U	-	N
**Benzodiazepines as first choice for older persons**	8 (8)	N	Sens	8.5 (6)	N	A	N	Y^f^
			Spec	8.5 (6)		A	N	Y^f^
**Cough and cold medications**	9 (1)	N	Sens	9 (2)	N	A	Y	Y
			Spec	-		-	-	-
**Ineffective drugs (such as selected nootropics) for Alzheimer disease**	9 (0)	N	Sens	9 (1)	N	A	Y	Y
			Spec	-		-	-	-
**Opioids for acute non-specific back pain**	7 (2)	Y	Sens	7 (7)	Y	A	N	N^f^
			Spec	7 (7)		A	N	N^f^
**Opioids for migraine or headache**	9 (3)	N	Sens	9 (8)	N	A	Y	Y
			Spec	-		-	-	-
**Diagnostic tests**								
**Bone mineral density testing at frequent intervals**	8 (9)	N	Sens	8.5 (2)	N	A	Y	Y
			Spec	8.5 (3)		A	N	Y^f^
**Colonoscopy for constipation**	7.5 (8)	Y	Sens	7 (8)	Y	A	N	N^f^
			Spec	7 (8)		A	N	N^f^
**EEG for headache**	6 (6)	N	Sens	7 (7)	N	A	N	Y^f^
			Spec	7 (4)		A	Y	Y
**Endometrial biopsy for investigation of infertility**	8 (2)	N	Sens	9 (4)	N	A	N	N^f^
			Spec	9 (4)		A	N	N^f^
**Gastroscopy for dyspepsia**	8 (7)	N	Sens	7.5 (3)	N	A	N	Y^f^
			Spec	7.5 (3)		A	N	Y^f^
**Imaging for acute non-specific back pain**	8 (6)	N	Sens	6 (5)	N	U	-	N
			Spec	7.5 (4)		A	N	Y^f^
**Imaging for migraine or headache**	7 (8)	N	Sens	6 (8)	N	U	-	N
			Spec	7 (2)		A	N	Y^f^
**Preoperative chest radiography prior to selected surgeries**	8 (8)	Y	Sens	8 (6)	Y	A	Y	Y
			Spec	8 (8)		A	Y	Y
**Preoperative stress testing prior to selected surgeries**	8.5 (2)	Y	Sens	7 (6)	Y	A	Y	Y
			Spec	8 (8)		A	Y	Y
**Stress echocardiography for detection of coronary artery disease in ACS**	8 (8)	Y	Sens	8 (8)	N	A	N	N^f^
			Spec	-		-	-	-
**Stress testing for stable coronary disease**	5 (8)	N	Sens	5 (7)	N	U	-	N
			Spec	7.5 (7)		A	N	N^f^
**Spirometry for known COPD**	9 (8)	N	Sens	8.5 (7)	N	A	N	N^f^
			Spec	8.5 (7)		A	N	N^f^
**Testing for group A streptococcal pharyngitis**	8 (2)	N	Sens	7 (1)	N	A	Y	Y
			Spec	6 (5)		U	-	N
**Free T3/T4 level testing for hypothyroidism**	8 (6)	N	Sens	9 (3)	N	A	N	Y^f^
			Spec	9 (0)		A	Y	Y
**Tumour marker testing without cancer diagnosis**	6 (8)	Y	Sens	3.5 (8)	Y	U	-	N
			Spec	8 (8)		A	Y	Y
**Screening**								
**Cancer screening for dialysis-dependent chronic kidney disease**	6 (7)	Y	Sens	3.5 (8)	Y	U	-	N
			Spec	8.0 (6)		A	N	Y^f^
**Colorectal cancer screening for older persons**	5 (6)	Y	Sens	5 (8)	Y	U	-	N
			Spec	6.5 (8)		A	N	N^f^
**Mammography screening in older women**	7 (6)	Y	Sens	-	-	-	-	-
			Spec	-		-	-	-
**Mammography screening in younger women**	2.5 (7)	Y	Sens	-	-	-	-	-
			Spec	-		-	-	-
**Treatment**								
**Abdominal hysterectomy for benign diseases**	9 (6)	N	Sens	6.5 (8)	N	A	N	N^f^
			Spec	8 (8)		A	N	N^f^
**Chemotherapy for cancer in the last months of life**	4 (8)	Y	Sens	2 (8)	Y	I	-	N
			Spec	7 (7)		A	N	Y^f^
**Electrotherapy for pressure ulcer**	8 (2)	N	Sens	5 (6)	N	U	-	N
			Spec	7 (2)		A	Y	Y
**ERC for calculus of bile duct or acute pancreatitis without cholangitis**	6 (6)	Y	Sens	8 (7)	N	A	Y	Y
			Spec	8 (7)		A	N	Y^f^
**Epidural steroid injections for low back pain**	9 (1)	N	Sens	8 (4)	N	A	N	N^f^
			Spec	7 (4)		A	N	N^f^
**Inhalation therapy for COPD without previously confirming the diagnosis by spirometry**	9 (3)	N	Sens	8 (2)	N	A	Y	Y
			Spec	9 (1)		A	Y	Y
**PTA of the renal artery or stenting for selected diagnoses**	7 (6)	N	Sens	8 (2)	N	A	Y	Y
			Spec	8 (3)		A	N	Y^f^
**Postoperative radiation therapy after radical prostatectomy**	8.5 (6)	N	Sens	4.5 (6)	N	U	-	N
			Spec	4.5 (6)		U	-	N
**Removal of gallbladder during bariatric surgery**	8 (6)	Y	Sens	7.5 (7)	N	A	N	N^f^
			Spec	-		-	-	-
**Retinal laser therapy or cryotherapy for asymptomatic lattice degeneration**	6.5 (1)	N	Sens	-	-	-	-	-
			Spec	-		-	-	-
**Spinal fusion for low back pain**	8.5 (4)	N	Sens	7.5 (5)	N	A	N	N^f^
			Spec	7.5 (5)		A	N	N^f^
**Surgery for vesicoureteral reflux**	5 (8)	N	Sens	4.5 (7)	N	U	-	N
			Spec	-		-	-	-
**Tube feeding via PEG for dementia in the last months of life**	8 (8)	Y	Sens	8 (7)	Y	A	N	N^f^
			Spec	-		-	-	-
**Unblocking nasolacrimal duct**	8 (2)	N	Sens	8 (1)	N	A	Y	Y
			Spec	7 (1)		A	Y	Y

Abbreviations: A = Appropriate; ACS = Acute Coronary Syndrome; COPD = Chronic Obstructive Pulmonary Disease; EEG = Electroencephalography; ERC = Endoscopic Retrograde Cholangiography; I = Inappropriate; N = No; PEG = Percutaneous Endoscopic Gastrostomy; PTA = Percutaneous Transluminal Angioplasty; Sens = Sensitive; Spec = Specific; U = Uncertain; Y = Yes.

^a^Round 1: In the first round, the representatives only rated the appropriateness of the sensitive definition to pre-assess if the indicators defined low-value healthcare services.

^b^Median: Appropriateness ratings of 1–3 were classified as inappropriate, ratings of 4–6 were classified as uncertain and ratings 7–9 were classified as appropriate. Indicators which fell exactly between the three-point boundaries (3.5 and 6.5) were included in the higher appropriateness category.

^c^Validity: Results were categorised as valid when at least seven experts rated the indicator.

^d^Agreement: Indicators were categorised “with agreement” when 80% of the ratings were in the same range as the observed median (1–3; 4–6; 7–9) and “with disagreement” when 90% of the ratings were within one of two extra wide regions (1–6 or 4–9).

^e^Inclusion: Indicators rated as appropriate were included. Those rated as appropriate but “with disagreement” were further revised and refined with the respective representatives and subsequently, either included or excluded. Indicators rated as inappropriate or uncertain were excluded.

^f^Appropriate indicators rated with “disagreement” were further discussed, revised and refined with the respective representatives and subsequently, either included or excluded.

## Discussion

The objective of the three-round modified Delphi panel described in this study was to facilitate the selection of indicators for measuring low-value healthcare services in German claims data. This selection process was based on indicators previously developed and published in international literature. However, only a few sources have employed expert panels to assess these indicators [19–21]. In our study, we evaluated the suitability of these indicators for measuring low-value care in German claims data and made necessary modifications. We engaged representatives from various Scientific Medical Societies and professional organisations to assess the indicators within the German context using a modified Delphi method, following the RAND/UCLA approach.

The selection of indicators in this study was generally constrained by data limitations within the German claims dataset and specific German coding practices. Germany’s statutory health insurance claims data primarily serve billing and reimbursement purposes. Consequently, aspects such as the accuracy of ICD coding in the outpatient sector are less emphasized and there is a lack of coding regulations [22]. In addition, German claims data lack clinical details, such as disease severity, prognostic factors, or laboratory results. These inherent data limitations reduced the number of initially identified indicators from 171 to 42 that were considered potentially measurable within the constraints of German claims data.

Following a thorough assessment by the representatives during the Delphi panel, the set of indicators was further refined to 24 appropriate indicators (comprising 17 sensitive and 20 specific definitions) for measuring low-value care in German claims data. Of these, agreement was reached on twelve sensitive and ten specific definitions. While the selected indicators allow for the measurement of a relatively small set of low-value healthcare services, studies suggest that low-value care extends beyond these services [8]. However, many of these indicators cannot be measured due to the limitations of German claims data, particularly the lack of information on disease symptoms or severity. For example, indicators such as “antipsychotics as first choice for dementia” or “stress testing for stable coronary disease” were excluded. Although the representatives agreed that these interventions could indicate low-value care, they determined that additional clinical information would be necessary to reliably differentiate between appropriate and low-value care.

Our findings parallel those of other research aimed at developing measures to assess low-value care, such as the studies by Chalmers et al. and Sprenger et al., who similarly identified only a small subset of low-value care items as measurable within claims data [3, 23]. While we screened 171 pre-developed indicators, Chalmers et al. screened 824 Choosing Wisely recommendations—rather than fully developed indicators—ultimately identifying only 17 (representing 15 services) that could be assessed in hospital claims data in Australia [3]. Similarly, Sprenger et al. initially identified 453 potentially low-value services in Austrian primary care, yet only 34 (7.5%) of these services could be quantified using claims data [23]. These results underscore a common challenge across healthcare systems: administrative data alone, often designed for billing rather than clinical depth, may lack the granularity needed to fully capture low-value care. Nonetheless, these measurable indicators can serve as conservative, population-based benchmarks, useful for regional and institutional comparisons.

Some indicators in our study were rated as inappropriate in their sensitive definitions as they were too broad to accurately identify low-value healthcare services. For example, the sensitive definition of “electrotherapy for pressure ulcer” was rated as inappropriate, as electrotherapy is considered low-value only for certain stages of pressure ulcers [24]. The corresponding specific definition, which focuses on electrotherapy for stage 1 pressure ulcers only, was regarded by the representatives as a more appropriate indicator of low-value care. Remarkably, only one specific definition -“testing for group A streptococcal pharyngitis”- received a lower rating compared to its corresponding sensitive definition. The representatives expressed uncertainty regarding its appropriateness due to the infrequent use of the excluding diagnostic code in practice.

In our study, we further revised and refined 15 sensitive definitions and 19 specific definitions in collaboration with the representatives after the second survey round. One concerning individuals with a cancer diagnosis who received chemotherapy in the last month before death (specific definition was initially rated with disagreement). After discussion and refinement with the respective representatives, we included it in our study. This approach is similar to the method presented by De Schreye et al., whose indicator served as the basis for ours. In their study, the indicator was adapted and accepted in the plenary discussion after the scoring round. However, there were some differences in the acceptance of the sensitive definition between our study and that of De Schreye et al. While our study excluded the sensitive definition (chemotherapy in the last three months before death) after the second survey round, it was accepted in De Schreye et al.’s scoring round. This disparity may be attributed to differences in the scope or criteria applied. Specifically, De Schreye et al. limited their indicator to new-line chemotherapy, while our study considered all types of chemotherapy [19].

In this study, the modified Delphi panel consisted of three rounds, including two survey ratings by the representatives and one online meeting in between. Niederberger and Spranger showed, in an overview of twelve systematic reviews of Delphi studies across different sectors in health sciences, that most Delphi panels are carried out in two to three rounds, depending on how a “Delphi round” is defined. As there are discrepancies in the definition of a “modified Delphi study”, some studies identified the classic Delphi method as the most commonly used, whereas other studies found the modified Delphi method to be dominant [25]. For example, one systematic review including studies describing the selection of healthcare quality indicators defined the modified Delphi method as Delphi rounds with a physical meeting of the experts, which was used in 62.8% of the analysed studies. Most of these studies used both a rating scale and open questions to review the indicators, applied the median score and percentage of agreement as consensus method, and provided quantitative feedback [14]. In these respects, the method we applied in this study is largely consistent with common practice.

Overall, the two survey rounds yielded high response rates, with 91.2% in round one and 95.0% in round three, respectively. These response rates are in line with or even slightly higher than those reported in previous studies [25]. The high engagement and participation of the representatives could be attributed to their official appointment by medical societies and federal associations, rather than being selected based on individual criteria. We decided to recruit the representatives through societies and associations as organisations involved in the selection process are more likely to approve the resulting indicators [16]. The sending of multiple reminders may have also contributed to a high response rate [26].

In the third round of the Delphi process, median ratings of sensitive indicator definitions showed varying trends compared to round one. Most indicators received lower ratings, indicating a shift towards a less favourable perception of their appropriateness. Other indicators maintained the same rating across both rounds, suggesting a consistent assessment of their appropriateness over time, while the minority received a higher rating in round three, implying an improvement in their perceived appropriateness.

We intended that each indicator would be rated by a variety of relevant specialist areas as variation in group composition–rather than homogeneous groups–may enhance discussion [27, 28]. In our study, the experts represented various medical specialties. The majority of the experts worked in both clinical practice and academia, with a higher proportion practising in the inpatient sector compared to the outpatient sector. We also included patient representatives to reflect their perspectives. The representatives engaged in commenting and discussing their respective indicators and we subsequently modified the indicators based on their input. The input primarily concerned information on coding practices in everyday healthcare as well as clinical background information on the indicators. However, the involvement of different specialities may have also led to a higher level of dispersion in the ratings of appropriateness. Other studies have shown that ratings may vary across specialties as experts who perform the procedure usually have higher mean ratings than other specialities [29]. In addition, ratings may vary between mixed- and single-specialty panels, because single-specialty panels often rate more indications as appropriate than multidisciplinary panels [30, 31]. The constructivist nature of the Delphi method does not regard this as a problem, though, as the constructivist approach considers that experts’ perspectives are constructed and further developed within a social context. Consequently, these perspectives are dependent on the situation, as opposed to critical rationalism, which seeks to attain universal or objective knowledge [13]. Thus, the constructivist epistemology leads to unique perspectives in the selection of indicators that need to be acknowledged when interpreting the results.

This study has several limitations. Firstly, previous studies used the Delphi method to develop indicators related to one medical field, such as indicators for emergency care [32, 33], glioma care [34] or palliative day services [35]. In contrast, our study did not only focus on indicators from one single aspect of care but identified a comprehensive number of indicators representing a large variety of medical fields. This heterogeneity may have led to disagreement, as the representatives did not necessarily share the same perspectives guiding their responses. Also, this approach posed challenges in terms of recruiting representatives and timing the panel discussion, as each representative had to rate a different set of indicators. Consequently, the participation rate in the online discussion was relatively low. Nevertheless, those who were unable to participate were given the opportunity to provide remarks on their reviewed indicators in advance, allowing their opinions to be considered despite their absence.

Secondly, only ten indicators received ratings from seven or more representatives. The selection process, which involved medical societies and federal associations nominating one representative (and one substitute) from one medical field, may have led to a lower number of representatives for some indicators. Previous studies have reported a wide range in the number of experts involved, from as few as three to as many as 731 [25], highlighting that the optimal panel size depends on the specific objectives of the Delphi study. It is generally recommended to have a sufficient number of experts to ensure diversity, while still enabling meaningful participation and discussion among all panel members [16]. In this study, our primary objective was to strengthen the operationalisation of the identified indicators through clinical expertise and to gain the approval of the medical societies for the results. We considered the relatively small numbers of representatives sufficient to achieve this objective.

Thirdly, the representatives’ ratings were influenced by several factors. These factors encompassed the information presented to them [36], which included the evidence supporting each indicator and the individualised graph-based document summarising all panel ratings that each representative received. By providing only the extreme options on the Likert scale (“disagree completely” and “agree completely”), experts likely had to interpret the intermediate levels themselves, which, along with the phrasing of the survey questions, may have impacted the assessment. Although the survey instrument used in this study underwent pilot testing, it might not have been possible to entirely eliminate this bias. Overall, the sensitive definitions of the indicators were rated lower than the specific definitions. This discrepancy could be attributed to the broader formulation of sensitive definitions which might have rendered them seemingly less suitable in direct contrast to the narrower and more precise definitions. Also, despite our efforts to manage the discussions effectively, the lack of anonymity in the second round may have allowed some degree of prominent voice bias, where more influential participants could potentially sway the group. However, by implementing anonymous ratings in the subsequent round, we aimed to minimise this risk.

Fourthly, while patient representatives were included for 16 indicators where no evidence-based guidelines were available, they were not involved in the assessment of 26 other indicators based on their prior contributions to the development of related guidelines in Germany. However, this exclusion may have limited the breadth of patient perspectives in our study, potentially overlooking important insights that could have further enriched the evaluation and selection of the final set of indicators. Future studies might benefit from incorporating patient input at all stages to ensure a more comprehensive representation of their views.

Fifthly, our specific definitions of the indicators were not formally assessed by the representatives in round one. Instead, round one focused on a pre-assessment, where representatives evaluated whether the indicators, in general, defined low-value care. During the subsequent online meeting, both the sensitive and specific definitions were thoroughly discussed. In round three, the representatives rated both definitions. Moreover, since both the sensitive and specific definitions were derived from established international literature, we consider the specific definitions robust enough to indicate low-value care.

Lastly, the representatives primarily had backgrounds in clinical practice and research and may therefore have had limited familiarity with the billing practices in everyday healthcare. We attempted to address this by providing comprehensive preparatory materials that included detailed explanations of the operationalisation of the low-value care indicators. In general, we did not record the number of codes that were directly transferable from those used in the international literature. Despite these efforts, this potential gap in familiarity could have influenced their assessment of the operationalisation of some indicators. This limitation also applies to the patient representatives.

It is important to recognise that indicators can only be formulated at the population level, as individual decisions regarding the provision of healthcare services are influenced by various factors unique to each patient. The Delphi method does not guarantee the attainment of the optimal judgment or conclusion [37]. Rather, its outcomes should serve as a starting point for raising concerns and stimulating constructive discussions. Indicators should be viewed as a tool to initiate conversations about the appropriateness of delivering specific healthcare services to individual patients [16].

The fact that only 24 out of 171 initially identified indicators were deemed appropriate for measuring low-value care in German claims data raises questions about the suitability of claims data alone for this purpose. Nevertheless, we believe that claims data remain valuable for establishing conservative, population-based benchmarks within the insured patient cohort, which can facilitate comparative analyses across regions and institutions. However, implementing such indicators in applied quality management will require extensive validation, potentially through chart reviews or other clinical data sources. At this stage, these indicators provide a foundation for further research on the determinants of low-value care and for estimating its structural and financial impacts within the healthcare system.

Data limitations could affect the precision of estimating the absolute extent of low-value care. To address these issues, it would be helpful to link German claims data with patient record information and to revise legal documentation requirements to include details beyond what is necessary for billing purposes, particularly concerning disease severity. Future research exploring the limitations in measuring certain indicators could offer valuable insights for enhancing the quality and utility of German claims data.

Despite these limitations, the selected indicators can still be valuable in practice. Measuring low-value care using claims data can provide insights into the prevalence, trends, and patterns of low-value care, enabling prioritisation of interventions and informing policy decisions [4, 7]. Strategies such as implementing clinical decision support systems, providing performance feedback to healthcare providers, and enhancing provider education have proven effective in reducing low-value care [38]. The indicators are especially valuable for benchmarking across regions, institutions, and trends, assuming that the limitations are consistent across the dataset. While the selected indicators in this study are tailored to suit the constraints of German claims data, they may also be applicable in countries encountering similar challenges, such as a missing temporal link between diagnoses and potential low-value healthcare services or quarterly billing information. However, it is important to note that these indicators are not meant to be final. Instead they will be continuously refined and discussed. This allows for ongoing quality improvements based on emerging evidence and expert input.

## Conclusions

The Delphi panel provided valuable expertise in selecting indicators to measure low-value care in German claims data, helping to identify potential healthcare services and areas for improvement. Avoiding overuse and spending resources only on healthcare services that benefit patients is of high political and societal interest, especially given the growing healthcare costs and limited personnel resources. These indicators enable the estimation of low-value healthcare services within healthcare systems and can inform strategies for improving the quality of care. Nevertheless, limitations of claims data restrict the full potential of measuring low-value care in Germany. Extending legal documentation requirements is necessary to allow for a more reliable estimation of a broader range of low-value care indicators. Our findings thus contribute to a broader understanding of low-value care measurement and provide a foundation for further studies on its structural and financial impacts.

## Supporting information

S1 Table Overview of the contacted Scientific Medical Societies and professional organisations. Abbreviations: ACS = Acute Coronary Syndrome; COPD = Chronic Obstructive Pulmonary Disease; EEG = Electroencephalography; ERC = Endoscopic Retrograde Cholangiography; PEG = Percutaneous Endoscopic Gastrostomy; PTA = Percutaneous Transluminal Angioplasty.
(DOCX)

S2 Table Initial list of indicators. Abbreviations: ACS = Acute Coronary Syndrome; ATC = Anatomical Therapeutic Chemical codes; COPD = Chronic Obstructive Pulmonary Disease; EBM = uniform value scale (“Einheitlicher Bewertungsmaßstab”) codes; EEG = Electroencephalography; ERC = Endoscopic Retrograde Cholangiography; ICD = International Classification of Diseases 10th revision codes; PEG = Percutaneous Endoscopic Gastrostomy; OPS = Operation and Procedure Classification System codes; PTA = Percutaneous Transluminal Angioplasty. ^a^: All indicators are defined for the use in ambulatory and hospital care, unless stated otherwise.
(DOCX)

S3 Table Number of respondents per indicator. Abbreviations: ACS = Acute Coronary Syndrome; COPD = Chronic Obstructive Pulmonary Disease; EEG = Electroencephalography; ERC = Endoscopic Retrograde Cholangiography; PEG = Percutaneous Endoscopic Gastrostomy; PTA = Percutaneous Transluminal Angioplasty.
(DOCX)

S4 Table Excluding diagnoses and services. (DOCX)

S1 Fig Evaluation example of a report after the first round. (TIF)

S1 File Evidence example of one indicator. (DOCX)

S2 File Medical specialties in the Delphi panel. (DOCX)

S1 Dataset All respondents per indicator. Abbreviations: ACS = Acute Coronary Syndrome; COPD = Chronic Obstructive Pulmonary Disease; EEG = Electroencephalography; ERC = Endoscopic Retrograde Cholangiography; N = No; NS = No Statement; PEG = Percutaneous Endoscopic Gastrostomy; PTA = Percutaneous Transluminal Angioplasty; Sens = Sensitive; Spec = Specific; U = Uncertain; Y = Yes. ^a^: Answer options range from 1 (= disagree completely) to 9 (= agree completely) or NS (= I cannot make a statement). ^b^: Due to confidentiality, answers are not shown when the number of respondents was n = 2.
(XLSX)
