# Supplementary material for: Selecting indicators for the measurement of low-value care using German claims data: A three-round modified Delphi panel
Source: PLoS One. 2025 Feb 18;20(2):e0314864. doi: 10.1371/journal.pone.0314864 (PMC11835324; doi:10.1371/journal.pone.0314864)
Supplement: S1 Table — Abbreviations: ACS = Acute Coronary Syndrome; COPD = Chronic Obstructive Pulmonary Disease; EEG = Electroencephalography; ERC = Endoscopic Retrograde Cholangiography; PEG = Percutaneous Endoscopic Gastrostomy; PTA = Percutaneous Transluminal Angioplasty. (DOCX) [file pone.0314864.s001.docx]

# **S1: Overview of the contacted Scientific Medical Societies and professional organisations**

| **Indicator** | **Scientific Medical Society / professional organisation** |
| --- | --- |
| **Pharmaceuticals** |  |
| **Acid blockers for uncomplicated gastroesophageal reflux** | German Society for Paediatric Gastroenterology and Nutrition |
|  | German Society of Paediatrics and Adolescent Medicine |
|  | Professional Association of Child and Adolescent Physicians |
| **Antibiotics for acute otitis media** | German Society for Paediatric Infectious Diseases |
|  | German Society of Oto-Rhino-Laryngology, Head and Neck Surgery |
|  | German Society of Paediatrics and Adolescent Medicine |
|  | Professional Association of Child and Adolescent Physicians |
| **Antibiotics for uncomplicated respiratory tract infections** | German Respiratory Society |
|  | German Society for Paediatric Infectious Diseases |
|  | German Society of General Practice and Family Medicine |
|  | German Society of Infectious Diseases |
|  | German Society of Internal Medicine |
|  | German Society of Oto-Rhino-Laryngology, Head and Neck Surgery |
|  | German Society of Paediatrics and Adolescent Medicine |
|  | Professional Association of Child and Adolescent Physicians |
| **Antipsychotics as first choice for dementia** | German Association for Psychiatry, Psychotherapy and Psychosomatics |
|  | German Geriatrics Society |
|  | German Neurological Society |
|  | German Society of Internal Medicine |
| **Benzodiazepines as first choice for older persons** | German Association for Psychiatry, Psychotherapy and Psychosomatics |
|  | German Geriatrics Society |
|  | German Neurological Society |
|  | German Society of Internal Medicine |
|  | German Society of Nursing Science |
| **Cough and cold medications** | German Society for Paediatric Infectious Diseases |
|  | German Society of Oto-Rhino-Laryngology, Head and Neck Surgery |
|  | German Society of Paediatrics and Adolescent Medicine |
|  | Professional Association of Child and Adolescent Physicians |
| **Ineffective drugs (such as selected nootropics) for Alzheimer disease** | German Association for Psychiatry, Psychotherapy and Psychosomatics |
|  | German Geriatrics Society |
|  | German Neurological Society |
|  | German Society of Internal Medicine |

| **Opioids for acute non-specific back pain** | German Neurological Society |
| --- | --- |
|  | German Pain Society |
|  | German Society for Orthopaedic and Orthopaedic Surgery |
|  | German Society for Orthopaedic and Trauma Surgery |
|  | German Society for Physical and Rehabilitation Medicine |
|  | German Society for Spine Surgery |
|  | German Society of Anaesthesiology and Intensive Care Medicine |
|  | German Society of General Practice and Family Medicine |
|  | Quality Commission "Pain Therapy" of the Association of Statutory Health Insurance Physicians Bremen |
| **Opioids for migraine or headache** | German Migraine and Headache Society |
|  | German Neurological Society |
|  | German Pain Society |
|  | German Society of Anaesthesiology and Intensive Care Medicine |
|  | German Society of General Practice and Family Medicine |
|  | Quality Commission "Pain Therapy" of the Association of Statutory Health Insurance Physicians Bremen |
| **Diagnostic tests** |  |
| **Bone mineral density testing at frequent intervals** | German Geriatrics Society |
|  | German Radiological Society |
|  | German Society for Orthopaedic and Orthopaedic Surgery |
|  | German Society for Orthopaedic and Trauma Surgery |
|  | German Society of Endocrinology |
|  | Orthopaedic Society for Osteology |
| **Colonoscopy for constipation** | German Cancer Society |
|  | German Society for Coloproctology |
|  | German Society for Gastroenterology, Digestive and Metabolic Diseases |
|  | German Society for Haematology and Medical Oncology |
|  | German Society of General Practice and Family Medicine |
|  | German Society of Internal Medicine |
|  | Patient representative |
|  | Professional Association of German Surgeons |
| **EEG for headache** | German Association for Psychiatry, Psychotherapy and Psychosomatics |
|  | German Migraine and Headache Society |
|  | German Neurological Society |
|  | German Society for Clinical Neurophysiology and Functional Imaging |
|  | German Society of General Practice and Family Medicine |

| **Endometrial biopsy for investigation of infertility** | German Society for Gynaecology and Obstetrics |
| --- | --- |
|  | German Society of Endocrinology |
|  | Patient representative |
|  | Professional Association of Gynaecologists |
| **Gastroscopy for dyspepsia** | German Society for Gastroenterology, Digestive and Metabolic Diseases |
|  | German Society of Internal Medicine |
| **Imaging for acute non-specific back pain** | German Neurological Society |
|  | German Radiological Society |
|  | German Society for Orthopaedic and Orthopaedic Surgery |
|  | German Society for Orthopaedic and Trauma Surgery |
|  | German Society for Physical and Rehabilitation Medicine |
|  | German Society for Spine Surgery |
|  | German Society of General Practice and Family Medicine |
| **Imaging for migraine or headache** | German Association for Psychiatry, Psychotherapy and Psychosomatics |
|  | German Migraine and Headache Society |
|  | German Neurological Society |
|  | German Radiological Society |
|  | German Society of General Practice and Family Medicine |
| **Preoperative chest radiography prior to selected surgeries** | German Cardiac Society |
|  | German Radiological Society |
|  | German Society for Gynaecology and Obstetrics |
|  | German Society for Orthopaedic and Orthopaedic Surgery |
|  | German Society for Orthopaedic and Trauma Surgery |
|  | German Society of Anaesthesiology and Intensive Care Medicine |
|  | German Society of General Practice and Family Medicine |
|  | German Society of Neurosurgery |
|  | German Society of Ophthalmology |
|  | Professional Association of German Surgeons |
|  | Professional Association of Gynaecologists |
| **Preoperative stress testing prior to selected surgeries** | German Cardiac Society |
|  | German Society for Gynaecology and Obstetrics |
|  | German Society for Orthopaedic and Orthopaedic Surgery |
|  | German Society for Orthopaedic and Trauma Surgery |
|  | German Society of Anaesthesiology and Intensive Care Medicine |
|  | German Society of General Practice and Family Medicine |
|  | German Society of Neurosurgery |
|  | German Society of Ophthalmology |
|  | Professional Association of German Surgeons |
|  | Professional Association of Gynaecologists |

| **Stress echocardiography for detection of coronary artery disease in ACS** | German Cardiac Society |
| --- | --- |
|  | German Geriatrics Society |
|  | German Society of General Practice and Family Medicine |
|  | German Society of Internal Medicine |
|  | Patient representative |
| **Stress testing for stable coronary disease** | German Cardiac Society |
|  | German Geriatrics Society |
|  | German Society of General Practice and Family Medicine |
|  | German Society of Internal Medicine |
| **Spirometry for known COPD** | German Geriatrics Society |
|  | German Respiratory Society |
|  | German Society of General Practice and Family Medicine |
|  | German Society of Internal Medicine |
| **Testing for group A streptococcal pharyngitis** | German Society for Paediatric Infectious Diseases |
|  | German Society of Oto-Rhino-Laryngology, Head and Neck Surgery |
|  | German Society of Paediatrics and Adolescent Medicine |
|  | Professional Association of Child and Adolescent Physicians |
| **Free T3/T4 level testing for hypothyroidism** | German Society of Endocrinology |
|  | German Society of General Practice and Family Medicine |
|  | German Society of Internal Medicine |
| **Tumour marker testing without cancer diagnosis** | German Cancer Society |
|  | German Society for Gastroenterology, Digestive and Metabolic Diseases |
|  | German Society for Gynaecology and Obstetrics |
|  | German Society for Haematology and Medical Oncology |
|  | German Society for Urology |
|  | German Society of General Practice and Family Medicine |
|  | Professional Association of Gynaecologists |
| **Screening** |  |
| **Cancer screening for dialysis-dependent chronic kidney disease** | German Cancer Society |
|  | German Geriatrics Society |
|  | German Society for Haematology and Medical Oncology |
|  | German Society for Nephrology |
|  | German Society of General Practice and Family Medicine |
|  | German Society of Internal Medicine |
|  | Patient representative |

| **Colorectal cancer screening for older persons** | German Cancer Society |
| --- | --- |
|  | German Geriatrics Society |
|  | German Society for Coloproctology |
|  | German Society for Gastroenterology, Digestive and Metabolic Diseases |
|  | German Society for Haematology and Medical Oncology |
|  | German Society of General Practice and Family Medicine |
|  | German Society of Internal Medicine |
|  | Professional Association of German Surgeons |
| **Mammography screening in older women** | German Cancer Society |
|  | German Geriatrics Society |
|  | German Radiological Society |
|  | German Society for Gynaecology and Obstetrics |
|  | German Society for Haematology and Medical Oncology |
|  | German Society of General Practice and Family Medicine |
|  | Patient representative |
|  | Professional Association of Gynaecologists |
| **Mammography screening in younger women** | German Cancer Society |
|  | German Radiological Society |
|  | German Society for Gynaecology and Obstetrics |
|  | German Society for Haematology and Medical Oncology |
|  | German Society of General Practice and Family Medicine |
|  | Patient representative |
|  | Professional Association of Gynaecologists |
| **Treatment** |  |
| **Abdominal hysterectomy for benign diseases** | German Cancer Society |
|  | German Society for Gynaecology and Obstetrics |
|  | German Society of Endocrinology |
|  | Patient representative |
|  | Professional Association of Gynaecologists |
| **Chemotherapy for cancer in the last months of life** | German Geriatrics Society |
|  | German Association for Palliative Medicine |
|  | German Association for Psychiatry, Psychotherapy and Psychosomatics |
|  | German Cancer Society |
|  | German Society for Haematology and Medical Oncology |
|  | German Society of Internal Medicine |
|  | German Society of Nursing Science |
|  | Patient representative |
|  | Quality Commission "Pain Therapy" of the German Association of Statutory Health Insurance Physicians Bremen |
| **Electrotherapy for pressure ulcer** | German Society of Dermatology |
|  | German Geriatrics Society |
|  | German Society of Internal Medicine |
|  | German Society of Nursing Science |
|  | Professional Association of German Surgeons |
| **ERC for calculus of bile duct or acute pancreatitis without cholangitis** | Professional Association of German Surgeons |
|  | German Geriatrics Society |
|  | German Radiological Society |
|  | German Society for Gastroenterology, Digestive and Metabolic Diseases |
|  | German Society of Internal Medicine |
| **Epidural steroid injections for low back pain** | German Pain Society |
|  | German Society for Orthopaedic and Orthopaedic Surgery |
|  | German Society for Orthopaedic and Trauma Surgery |
|  | German Society for Physical and Rehabilitation Medicine |
|  | German Society for Spine Surgery |
|  | German Society of Anaesthesiology and Intensive Care Medicine |
|  | Quality Commission "Pain Therapy" of the German Association of Statutory Health Insurance Physicians Bremen |
| **Inhalation therapy for COPD without previously confirming the diagnosis by spirometry** | German Society for General Practice and Family Medicine |
|  | German Geriatrics Society |
|  | German Respiratory Society |
|  | German Society of Internal Medicine |
| **PTA of the renal artery or stenting for selected diagnoses** | German Society for Angiology |
|  | German Society for Nephrology |
|  | German Society for Vascular Surgery and Vascular Medicine |
|  | German Society of Internal Medicine |
| **Postoperative radiation therapy after radical prostatectomy** | German Cancer Society |
|  | German Society for Haematology and Medical Oncology |
|  | German Society for Radiation Oncology |
|  | German Society for Urology |
| **Removal of gallbladder during bariatric surgery** | German Society for Gastroenterology, Digestive and Metabolic Diseases |
|  | German Society of Internal Medicine |
|  | Professional Association of German Surgeons |
|  | Working Group on Obesity Surgery and Metabolic Surgery |
| **Retinal laser therapy or cryotherapy for asymptomatic lattice degeneration** | German Society of Ophthalmology |
|  | Patient representative |

| **Spinal fusion for low back pain** | German Society for Orthopaedic and Orthopaedic Surgery |
| --- | --- |
|  | German Society for Orthopaedic and Trauma Surgery |
|  | German Society for Spine Surgery |
|  | German Society of Neurosurgery |
|  | German Society of Paediatric Surgery |
|  | Patient representative |
| **Surgery for vesicoureteral reflux** | German Society of Paediatric Surgery |
|  | German Society of Paediatrics and Adolescent Medicine |
|  | Professional Association of Child and Adolescent Physicians |
| **Tube feeding via PEG for dementia in the last months of life** | German Geriatrics Society |
|  | German Association for Palliative Medicine |
|  | German Association for Psychiatry, Psychotherapy and Psychosomatics |
|  | German Neurological Society |
|  | German Society for Gastroenterology, Digestive and Metabolic Diseases |
|  | German Society of Internal Medicine |
|  | German Society of Nursing Science |
|  | Quality Commission "Pain Therapy" of the German Association of Statutory Health Insurance Physicians Bremen |
| **Unblocking nasolacrimal duct** | German Society of Ophthalmology |
|  | German Society of Oto-Rhino-Laryngology, Head and Neck Surgery |
|  | German Society of Paediatrics and Adolescent Medicine |
|  | Professional Association of Child and Adolescent Physicians |
