# Supplementary material for: Selecting indicators for the measurement of low-value care using German claims data: A three-round modified Delphi panel
Source: PLoS One. 2025 Feb 18;20(2):e0314864. doi: 10.1371/journal.pone.0314864 (PMC11835324; doi:10.1371/journal.pone.0314864)
Supplement: S2 Table — Abbreviations: ACS = Acute Coronary Syndrome; ATC = Anatomical Therapeutic Chemical codes; COPD = Chronic Obstructive Pulmonary Disease; EBM = uniform value scale (“Einheitlicher Bewertungsmaßstab”) codes; EEG = Electroencephalography; ERC = Endoscopic Retrograde Cholangiography; ICD = International Classification of Diseases 10th revision codes; PEG = Percutaneous Endoscopic Gastrostomy; OPS = Operation and Procedure Classification System codes; PTA = Percutaneous Transluminal Angioplasty. a: All indicators are defined for the use in ambulatory and hospital care, unless stated otherwise. (DOCX) [file pone.0314864.s002.docx]

# **S4: Initial list of indicators**

| **Indicator** | **Numerator^a^** | | **Denominator** | | **Source** |
| --- | --- | --- | --- | --- | --- |
| **Pharmaceuticals** |  | |  | |  |
| **Acid blockers for uncomplicated gastroesophageal reflux** | Sensitive: | Infants (< 1 year) who received prescriptions for oral H2 blockers or oral proton pump inhibitors in ambulatory care.  Exclusion: - | Population: | Infants (< 1 year). | [1, 2] |
|  | Specific: | Infants (< 1 year) who received prescriptions for oral H2 blockers or oral proton pump inhibitors in ambulatory care.  Exclusion: **Excluded diagnoses are listed below**. | Service: | Infants (< 1 year) who received prescriptions for oral H2 blockers or oral proton pump inhibitors. |  |
| **Antibiotics for acute otitis media** | Sensitive: | Children (2-12 years) with a diagnosis of acute otitis media who received prescriptions for systemic antibiotics in ambulatory care.  Exclusion: Excluded diagnoses are listed below. | Population: | Children (2-12 years) with a diagnosis of acute otitis media. | [3] |
|  | Specific: | Children (2-12 years) with a diagnosis of acute otitis media who received prescriptions for systemic antibiotics in ambulatory care.  Exclusion: Excluded diagnoses **and services** are listed below. | Service: | Children (2-12 years) who received prescriptions for systemic antibiotics. |  |
| **Antibiotics for uncomplicated respiratory tract infections** | Sensitive: | Persons (> 0 years) with a diagnosis of uncomplicated respiratory tract infections (including bronchitis, sinusitis, pharyngitis) who received prescriptions for systemic antibiotics in ambulatory care.  Exclusion: Excluded diagnoses are listed below. | Population: | Persons (> 0 years) with a diagnosis of uncomplicated respiratory tract infections (including bronchitis, sinusitis, pharyngitis). | [1–3] |
|  | Specific: | Persons (> 0 years) with a diagnosis of uncomplicated respiratory tract infections (including bronchitis, sinusitis, pharyngitis) who received prescriptions for systemic antibiotics in ambulatory care.  Exclusion: Excluded diagnoses **and services** are listed below. | Service: | Persons (> 0 years) who received prescriptions of systemic antibiotics. |  |
| **Antipsychotics as first choice for dementia** | Sensitive: | Persons (≥ 18 years) with a dementia diagnosis who received prescriptions for antipsychotics in ambulatory care.  Exclusion: Excluded diagnoses are listed below. | Population: | Persons (≥ 18 years) with a dementia diagnosis. | [4–7] |
|  | Specific: | Persons (≥ 18 years) with a dementia diagnosis who received prescriptions for antipsychotics in **two consecutive quarters** in ambulatory care.  Exclusion: Excluded diagnoses are listed below. | Service: | Persons (≥ 18 years) who received prescriptions for antipsychotics. |  |
| **Benzodiazepines as first choice for older persons** | Sensitive: | Persons (≥ 65 years) who received prescriptions for benzodiazepines in ambulatory care.  Exclusion: - | Population: | Persons (≥ 65 years). | [4] |
|  | Specific: | Persons (≥ 65 years) with a **diagnosis of sleep disorders, delirium or schizophrenia or symptoms and signs involving emotional state** who received prescriptions for benzodiazepines in ambulatory care.  Exclusion: **Excluded diagnoses are listed below**. | Service: | Persons (≥ 65 years) who received prescriptions for benzodiazepines. |  |
| **Cough and cold medications** | Sensitive: | Children (< 6 years) who received prescriptions for cough and cold medications in ambulatory care.  Exclusion: - | Population: | Children (< 6 years). | [1, 2] |
|  | Specific: | - | Service: | Children (< 6 years) who received prescriptions for cough and cold medications. |  |
| **Ineffective drugs (such as selected nootropics) for Alzheimer disease** | Sensitive: | Persons (≥ 18 years) with a diagnosis of Alzheimer disease who received prescriptions for ineffective drugs (such as selected nootropics) in ambulatory care.  Exclusion: - | Population: | Persons (≥ 18 years) with a diagnosis of Alzheimer disease. | [8] |
|  | Specific: | - | Service: | Persons (≥ 18 years) who received prescriptions for ineffective medications (such as selected nootropics). |  |
| **Opioids for acute non-specific back pain** | Sensitive: | Persons (≥ 18 years) with a diagnosis of acute non-specific low back pain (dorsalgia) who received prescriptions for opioids in ambulatory care.  Exclusion: Excluded diagnoses are listed below. | Population: | Persons (≥ 18 years) with a diagnosis of acute non-specific back pain (dorsalgia). | [9] |
|  | Specific: | Persons (≥ 18 years) with a diagnosis of acute non-specific low back pain (**low back pain, other dorsalgia or dorsalgia, unspecified**) who received prescriptions for opioids in ambulatory care.  Exclusion: Excluded diagnoses are listed below. | Service: | Persons (≥ 18 years) who received prescriptions for opioids. |  |
| **Opioids for migraine or headache** | Sensitive: | Persons (≥ 18 years) with a diagnosis of migraine or headache who received prescriptions for opioids in ambulatory care.  Exclusion: Excluded diagnoses and services are listed below. | Population: | All persons (≥ 18 years) with a diagnosis of migraine or headache. | [5, 9–12] |
|  | Specific: | - | Service: | Persons (≥ 18 years) who received prescriptions for opioids. |  |
| **Diagnostic tests** |  |  |  |  |  |
| **Bone mineral density testing at frequent intervals** | Sensitive: | Persons (≥ 18 years) who received bone mineral density testing within the first two years after bone mineral density testing.  Exclusion: - | Population: | Persons (≥ 18 years). | [13, 14] |
|  | Specific: | Persons (≥ 18 years) **with a diagnosis of osteoporosis** who received bone mineral density testing within the first two years after bone mineral density testing.  Exclusion: **Excluded diagnoses and services are listed below**. | Service: | Persons (≥ 18 years) who received bone mineral density testing. |  |
| **Colonoscopy for constipation** | Sensitive: | Persons (< 50 years) with a diagnosis of constipation who received colonoscopy.  Exclusion: Excluded diagnose are listed below. | Population: | Persons (< 50 years) with a diagnosis of constipation. | [15, 16] |
|  | Specific: | Persons (< 50 years) with a diagnosis of constipation who received colonoscopy **in the past** **four quarters**.  Exclusion: Excluded diagnoses are listed below. | Service: | Persons (< 50 years) who received colonoscopy. |  |
| **EEG for headache** | Sensitive: | Persons (≥ 18 years) with a diagnosis of headache who received an EEG.  Exclusion: Excluded diagnoses are listed below. | Population: | Persons (≥ 18 years) with a diagnosis of headache. | [13, 17] |
|  | Specific: | Persons (≥ 18 years) with a diagnosis of headache who received an EEG.  Exclusion: Excluded diagnoses are listed below (**different from sensitive**). | Service: | Persons (≥ 18 years) who received an EEG. |  |
| **Endometrial biopsy for investigation of infertility** | Sensitive: | Women (18 - 50 years) with a diagnosis of infertility who received an endometrial biopsy.  Exclusion: Excluded diagnoses are listed below. | Population: | Women (18 - 50 years) with a diagnosis of infertility. | [15, 16] |
|  | Specific: | Women (18 - 50 years) with a **primary** **diagnosis of infertility** who received an endometrial biopsy.  Exclusion: Excluded diagnoses are listed below. | Service: | Women (18 - 50 years) who received an endometrial biopsy. |  |
| **Gastroscopy for dyspepsia** | Sensitive: | Persons (18-54 years) with a diagnosis of dyspepsia who received a gastroscopy.  Exclusion: Excluded diagnoses are listed below. | Population: | Persons (18-54 years) with a diagnosis of dyspepsia. | [15, 16] |
|  | Specific: | Persons (18-54 years) with a diagnosis of dyspepsia who received gastroscopy.  Exclusion: Excluded diagnoses are listed below (**different from sensitive**). | Service: | Persons (18-54 years) who received gastroscopy. |  |
| **Imaging for acute non-specific back pain** | Sensitive: | Persons (≥ 18 years) with an incident diagnosis of acute non-specific back pain (dorsalgia) who received radiologic imaging in ambulatory care and who had a bill for radiologic lump-sums or a visit to a radiologist.  Exclusion: Excluded diagnoses are listed below. | Population: | Persons (≥ 18 years) with an incident diagnosis of non-specific back pain (dorsalgia). | [18] |
|  | Specific: | Persons (≥ 18 years) with an incident diagnosis of acute non-specific back pain (**low back pain, other dorsalgia or dorsalgia, unspecified**) who received radiologic imaging in ambulatory care and who had a bill for radiologic lump-sums or a visit to a radiologist.  Exclusion: Excluded diagnoses are listed below. | Service: | Persons (≥ 18 years) who received radiologic imaging and who had a bill for radiologic lump-sums or a visit to a radiologist. |  |
| **Imaging for migraine or headache** | Sensitive: | Persons (18-50 years) with a diagnosis of migraine or headache who received imaging of the head.  Exclusion: Excluded diagnoses are listed below. | Population: | Persons (18-50 years) with a diagnosis of migraine or headache. | [10, 12, 13, 19] |
|  | Specific: | Persons (18-50 years) with a **known** diagnosis of migraine or headache who received imaging of the head.  Exclusion: Excluded diagnoses are listed below (**different from sensitive**). **No emergency care**. | Service: | Persons (18-50 years) who received imaging of the head. |  |
| **Preoperative chest radiography prior to selected surgeries** | Sensitive: | Persons (≥ 18 years) who received chest radiography within 30 days prior to selected low-risk non-cardiothoracic surgical procedures or anaesthesia in ambulatory care.  Exclusion: Excluded diagnoses and services are listed below. | Population: | Persons (≥18 years) who received selected low-risk non-cardiothoracic surgical procedures. | [13, 14, 19–25] |
|  | Specific: | Persons (≥ 18 years) who received chest radiography within 30 days prior to selected low-risk non-cardiothoracic surgical procedures or anaesthesia in ambulatory care.  Exclusion: Excluded diagnoses and services are listed below (**different from sensitive**). | Service: | Persons (≥18 years) who received chest radiography. |  |
| **Preoperative stress testing prior to selected surgeries** | Sensitive: | Persons (≥ 65 years) who received stress testing within 30 days prior to selected low-risk non-cardiothoracic surgical procedures.  Exclusion: Excluded diagnoses and services are listed below. | Population: | Persons (≥ 65 years) who received selected low-risk non-cardiothoracic surgical procedures. | [5, 13, 14, 20, 21] |
|  | Specific: | Persons (≥ 65 years) who received stress testing within 30 days prior to selected low-risk non-cardiothoracic surgical procedures **in ambulatory care**.  Exclusion: Excluded diagnoses and services are listed below. | Service: | Persons (≥ 65 years) who received stress testing. |  |
| **Stress echocardiography for detection of coronary artery disease in ACS** | Sensitive: | Persons (≥ 18 years) with a diagnosis associated with ACS who received stress echocardiography in hospital care.  Exclusion: - | Population: | Persons (≥ 18 years) with a diagnosis associated with ACS. | [23–25] |
|  | Specific: | - | Service: | Persons (≥ 18 years) who received stress echocardiography. |  |
| **Stress testing for stable coronary disease** | Sensitive: | Persons (≥ 18 years) with a diagnosis of ischaemic heart disease or angina pectoris who received stress testing.  Exclusion: - | Population: | Persons (≥ 18 years) with a diagnosis of ischaemic heart disease or angina pectoris. | [13, 14] |
|  | Specific: | Persons (≥ 18 years) with a diagnosis of **previous myocardial infarction** who received **routine** stress testing **in ambulatory care**.  Exclusion: - | Service: | Persons (≥ 18 years) who received stress testing. |  |
| **Spirometry for known COPD** | Sensitive: | Persons (≥ 18 years) with a known COPD diagnosis who received a spirometry.  Exclusion: - | Population: | Persons (≥ 18 years) with a known COPD diagnosis. | [26] |
|  | Specific: | Persons (≥ 18 years) with a known COPD diagnosis **with acute exacerbation** who received a spirometry.  Exclusion: - | Service: | Persons (≥ 18 years) who received a spirometry. |  |
| **Testing for group A streptococcal pharyngitis** | Sensitive: | Children (< 3 years) who received testing for group A streptococcal pharyngitis in ambulatory care.  Exclusion: - | Population: | Children (< 3 years). | [1, 2] |
|  | Specific: | Children (< 3 years) who received testing for group A streptococcal pharyngitis in ambulatory care.  Exclusion: **Excluded diagnoses are listed below**. | Service: | Children (< 3 years) who received testing for group A streptococcal pharyngitis. |  |
| **Free T3/T4 level testing for hypothyroidism** | Sensitive: | Persons (≥ 18 years) with a known diagnosis of hypothyroidism who received free T3 or **free T4 level** testing in ambulatory care.  Exclusion: - | Population: | Persons (≥ 18 years) with a known diagnosis of hypothyroidism. | [14] |
|  | Specific: | Persons (≥18 years) with a known diagnosis of hypothyroidism who received free T3 level testing in ambulatory care.  Exclusion: **Excluded diagnoses are listed below**. | Service: | Persons (≥ 18 years) who received free T3 or free T4 level testing. |  |
| **Tumour marker testing without cancer diagnosis** | Sensitive: | Persons (≥ 18 years) who received tumour marker testing in ambulatory care.  Exclusion: Excluded diagnoses are listed below. | Population: | Persons (≥ 18 years). | [27] |
|  | Specific: | Persons (≥ 18 years) who received tumour marker testing in ambulatory care.  Exclusion: Excluded diagnoses are listed below (**different from sensitive**). | Service: | Persons (≥ 18 years) who received tumour marker testing. |  |
| **Screening** |  |  |  |  |  |
| **Cancer screening for dialysis-dependent chronic kidney disease** | Sensitive: | Persons (≥ 75 years) with a diagnosis of stage 5 chronic kidney disease who received cancer screening.  Exclusion: - | Population: | Persons (≥ 75 years) with a diagnosis of stage 5 chronic kidney disease. | [13] |
|  | Specific: | Persons (≥ 75 years) with a diagnosis of stage 5 chronic kidney disease **receiving dialysis services** who received cancer screening.  Exclusion: - | Service: | Persons (≥ 75 years) who received cancer screening. |  |
| **Colorectal cancer screening for older persons** | Sensitive: | Persons (≥ 80 years) who received colorectal cancer screening in ambulatory care.  Exclusion: - | Population: | Persons (≥ 80 years). | [13, 14] |
|  | Specific: | Persons (≥ 80 years) who received colorectal cancer screening in ambulatory care.  Exclusion: **Excluded diagnoses are listed below**. | Service: | Persons (≥ 80 years) who received colorectal cancer screening. |  |
| **Mammography screening in older women** | Sensitive: | Women (≥ 80 years) who received a mammogram.  Exclusion: - | Population: | Women (≥ 80 years). | [28] |
|  | Specific: | Women (≥ 80 years) who received a mammogram.  Exclusion: **Excluded diagnoses are listed below**. | Service: | Women (≥ 80 years) who received a mammogram. |  |
| **Mammography screening in younger women** | Sensitive: | Women (40-49 years) who received a mammogram.  Exclusion: - | Population: | Women (40-49 years). | [20, 29] |
|  | Specific: | Women (40-49 years) who received a mammogram.  Exclusion: **Excluded diagnoses and services are listed below**. | Service: | Women (40-49 years) who received a mammogram. |  |
| **Treatment** |  |  |  |  |  |
| **Abdominal hysterectomy for benign diseases** | Sensitive: | Women (≥ 18 years) who received an abdominal hysterectomy in hospital care.  Exclusion: Excluded diagnoses and services are listed below. | Population: | Women (≥18 years). | [16] |
|  | Specific: | Women (≥ 18 years) who received an abdominal hysterectomy in hospital care.  Exclusion: Excluded diagnoses and services are listed below (**different from sensitive**). | Service: | Women (≥ 18 years) who received a hysterectomy (laparoscopic, vaginal or abdominal). |  |
| **Chemotherapy for cancer in the last months of life** | Sensitive: | Persons (≥ 18 years) with a cancer diagnosis who received chemotherapy in the last three months prior to death.  Exclusion: - | Population: | Persons (≥ 18 years) who died with a cancer diagnosis. | [30–33] |
|  | Specific: | Persons (≥ 18 years) with a cancer diagnosis who received chemotherapy **in the last month** prior to death.  Exclusion: - | Service: | Persons (≥ 18 years) who died and received chemotherapy. |  |
| **Electrotherapy for pressure ulcer** | Sensitive: | Persons (> 0 years) with a diagnosis of pressure ulcer who received electrotherapy.  Exclusion: - | Population: | Persons (> 0 years) with a diagnosis of pressure ulcer. | [16] |
|  | Specific: | Persons (> 0 years) with a diagnosis of **stage 1 pressure ulcer** who received electrotherapy.  Exclusion: **Excluded diagnoses are listed below**. | Service: | Persons (> 0 years) who received electrotherapy. |  |
| **ERC for calculus of bile duct or acute pancreatitis without cholangitis** | Sensitive: | Persons (≥ 18 years) with a diagnosis of calculus of bile duct or acute pancreatitis who received ERC.  Exclusion: Excluded diagnoses are listed below. | Population: | Persons (≥ 18 years) with a diagnosis of calculus of bile duct or acute pancreatitis. | [16] |
|  | Specific: | Persons (≥ 18 years) with a diagnosis of calculus of bile duct or **biliary** acute pancreatitis who received ERC.  Exclusion: Excluded diagnoses are listed below. **No emergency care.** | Service: | Persons (≥ 18 years) who received ERC. |  |
| **Epidural steroid injections for low back pain** | Sensitive: | Persons (≥ 18 years) with a diagnosis of low back pain who received an epidural steroid injection in ambulatory care.  Exclusion: Excluded diagnoses are listed below. | Population: | Persons (≥ 18 years) with a diagnosis of low back pain. | [15, 16] |
|  | Specific: | Persons (≥ 18 years) with a diagnosis of low back pain who received an epidural steroid injection **in the past four quarters** in ambulatory care.  Exclusion: Excluded diagnoses are listed below. | Service: | Persons (≥ 18 years) who received an epidural steroid injection. |  |
| **Inhalation therapy for COPD without previously confirming the diagnosis by spirometry** | Sensitive: | Persons (≥ 18 years) with an incident COPD diagnosis without previously confirming the diagnosis by spirometry in the same quarter who received inhalation therapy in ambulatory care.  Exclusion: Excluded services are listed below. | Population: | Persons (≥ 18 years) with an incident COPD diagnosis. | [26] |
|  | Specific: | Persons (≥ 18 years) with an incident COPD diagnosis without previously confirming the diagnosis by spirometry in the same **or past quarter** who received inhalation therapy in ambulatory care.  Exclusion: Excluded services are listed below. | Service: | Persons (≥ 18 years) who received inhalation therapy. |  |
| **PTA of the renal artery or stenting for selected diagnoses** | Sensitive: | Persons (≥ 18 years) with a diagnosis of renovascular hypertension, atherosclerosis of renal artery, hypertensive kidney disease or **hypertensive heart and kidney disease** who received PTA of the renal artery or stenting in hospital care.  Exclusion: Excluded diagnoses are listed below. | Population: | Persons (≥ 18 years) with a diagnosis of renovascular hypertension, atherosclerosis of renal artery, hypertensive kidney disease, or hypertensive heart and kidney disease. | [16] |
|  | Specific: | Persons (≥ 18 years) with a diagnosis of renovascular hypertension or atherosclerosis of renal artery who received PTA of the renal artery or stenting in hospital care.  Exclusion: Excluded diagnoses are listed below. | Service: | Persons (≥ 18 years) who received PTA of the renal artery or stenting. |  |
| **Postoperative radiation therapy after radical prostatectomy** | Sensitive: | Men (≥ 18 years) who received radiation therapy in the first six weeks after radical prostatectomy in ambulatory care.  Exclusion: - | Population: | Men (≥ 18 years) after radical prostatectomy. | [16] |
|  | Specific: | Men (≥ 18 years) **with a diagnosis of prostate cancer** who received radiation therapy in the first six weeks after radical prostatectomy in ambulatory care.  Exclusion: - | Service: | Men (≥ 18 years) who received radiation therapy. |  |
| **Removal of gallbladder during bariatric surgery** | Sensitive: | Persons (≥ 18 years) who received cholecystectomy during bariatric surgery in hospital care.  Exclusion: Excluded diagnoses are listed below. | Population: | Persons (≥ 18 years) with a diagnosis of adiposity. | [15, 16] |
|  | Specific: | - | Service: | Persons (≥ 18 years) who received bariatric surgery. |  |
| **Retinal laser therapy or cryotherapy for asymptomatic lattice degeneration** | Sensitive: | Persons (≥ 18 years) with a diagnosis of lattice degeneration who received retinal laser therapy or cryotherapy.  Exclusion: Excluded diagnoses are listed below. | Population: | Persons (≥ 18 years) with a diagnosis of a lattice degeneration. | [15, 16] |
|  | Specific: | Persons (≥ 18 years) with a diagnosis of lattice degeneration who received retinal laser therapy or cryotherapy **in the past four quarters**.  Exclusion: Excluded diagnoses are listed below. | Service: | Persons (≥ 18 years) who received retinal laser therapy or cryotherapy. |  |
| **Spinal fusion for low back pain** | Sensitive: | Persons (≥ 18 years) with a diagnosis of low back pain who received a spinal fusion in hospital care.  Exclusion: Excluded diagnoses are listed below. | Population: | Persons (≥ 18 years) with a diagnosis of low back pain. | [15, 16] |
|  | Specific: | Persons (≥ 18 years) with a diagnosis of low back pain who received a spinal fusion **in the past four quarters** in hospital care.  Exclusion: Excluded diagnoses are listed below. | Service: | Persons (≥18 years) who received a spinal fusion. |  |
| **Surgery for vesicoureteral reflux** | Sensitive: | Children (< 11 years) who received a surgery for vesicoureteral reflux in hospital care.  Exclusion: - | Population: | Children (< 11 years) with a diagnosis of vesicoureteral reflux. | [16] |
|  | Specific: | - | Service: | Children (< 11 years) who received a surgery for vesicoureteral reflux. |  |
| **Tube feeding via PEG for dementia in the last months of life** | Sensitive: | Persons (≥ 18 years) with a dementia diagnosis who had a PEG insertion in the last three months prior to death.  Exclusion: - | Population: | Persons (≥ 18 years) who died with a dementia diagnosis. | [30–33] |
|  | Specific: | - | Service: | Persons (≥ 18 years) who had a PEG insertion. |  |
| **Unblocking nasolacrimal duct** | Sensitive: | Infants (< 1 year) who received probing of nasolacrimal ducts in hospital care.  Exclusion: - | Population: | Infants (< 1 year). | [15, 16] |
|  | Specific: | Infants (< 1 year) **with a diagnosis of inflammation or stenosis** who received probing of nasolacrimal ducts in hospital care.  Exclusion: - | Service: | Infants (< 1 year) who received probing of nasolacrimal ducts. |  |

# **Excluding diagnoses and services**

| **Indicator** | **Excluding services and diagnoses** | | **Sensitive** | **Specific** |
| --- | --- | --- | --- | --- |
| **Pharmaceuticals** | | | | |
| **Acid blockers for uncomplicated gastroesophageal reflux** | ***ICD*** |  |  |  |
|  | K20 | Oesophagitis |  | x |
|  | K25 | Gastric ulcer |  | x |
|  | K26 | Duodenal ulcer |  | x |
|  | K27 | Peptic ulcer, site unspecified |  | x |
|  | K28 | Gastrojejunal ulcer |  | x |
|  | K29 | Gastritis and duodenitis |  | x |
|  | K92.0 | Haematemesis |  | x |
|  | K92.8 | Other specified diseases of digestive system |  | x |
|  | P05.0 | Light for gestational age |  | x |
|  | P28.3 | Primary sleep apnea of newborn |  | x |
|  | P28.4 | Other apnea of newborn |  | x |
|  | P54.3 | Other neonatal gastrointestinal haemorrhage |  | x |
|  | P92.3 | Underfeeding of newborn |  | x |
|  | R06.8 | Other and unspecified abnormalities of breathing |  | x |
|  | R45.4 | Irritability and anger |  | x |
|  | R62 | Lack of expected normal physiological development |  | x |
|  | R63.4 | Abnormal weight loss |  | x |
|  | R68.1 | Nonspecific symptoms peculiar to infancy |  | x |
| **Antibiotics for acute otitis media** | ***ICD*** |  |  |  |
|  | A49 | Bacterial infection of unspecified site | x | x |
|  | A15 – A19 | Tuberculosis | x | x |
|  | A20 – A28 | Certain zoonotic bacterial diseases | x | x |
|  | B90.8 | Sequelae of tuberculosis of other organs | x | x |
|  | B90.9 | Sequelae of respiratory and unspecified tuberculosis | x | x |
|  | D80 – D89 | Certain disorders involving the immune mechanism | x | x |
|  | D90 | Immune comprise due to radiation, chemotherapy or other immunosuppressive measures | x | x |
|  | J02.0 | Streptococcal pharyngitis | x | x |
|  | J03.0 | Streptococcal tonsillitis | x | x |
|  | J13 | Pneumonia due to Streptococcus pneumoniae | x | x |
|  | J14 | Pneumonia due to Haemophilus influenzae | x | x |
|  | J15 | Bacterial pneumonia, not elsewhere classified | x | x |
|  | J16.0 | Chlamydial pneumonia | x | x |
|  | J17.0 | Pneumonia in bacterial diseases classified elsewhere | x | x |
|  | J20.0 | Acute bronchitis due to Mycoplasma pneumoniae | x | x |
|  | J20.1 | Acute bronchitis due to Haemophilus influenzae | x | x |
|  | J20.2 | Acute bronchitis due to streptococcus | x | x |
|  | J41 | Simple and mucopurulent chronic bronchitis | x | x |
|  | H60.0 | Abscess of external ear | x | x |
|  | H60.1 | Cellulitis of external ear | x | x |
|  | H60.3 | Other infective otitis externa | x | x |
|  | H60.4 | Cholesteatoma of external ear | x | x |
|  | H65.2 | Chronic serous otitis media | x | x |
|  | H65.3 | Chronic mucoid otitis media | x | x |
|  | H65.4 | Other chronic nonsuppurative otitis media | x | x |
|  | H66.0 | Acute suppurative otitis media | x | x |
|  | Q18 | Other congenital malformations of face and neck | x | x |
|  | Q87.0 | Congenital malformation syndromes predominantly affecting facial appearance | x | x |
|  | R50 | Fever of other and unknown origin | x | x |
|  | Z96.2 | Presence of otological and audiological implants | x | x |
|  | ***ATC*** |  |  |  |
|  | J01CA04 | Amoxicillin |  | x |
|  | J01FA | Macrolides |  | x |
| **Antibiotics for uncomplicated respiratory tract infections** | ***ICD*** |  |  |  |
|  | A49 | Bacterial infection of unspecified site | x | x |
|  | A15 – A19 | Tuberculosis | x | x |
|  | A20 – A28 | Certain zoonotic bacterial diseases | x | x |
|  | B90.8 | Sequelae of tuberculosis of other organs | x | x |
|  | B90.9 | Sequelae of respiratory and unspecified tuberculosis | x | x |
|  | D80 – D89 | Certain disorders involving the immune mechanism | x | x |
|  | D90 | Immune compromise due to radiation, chemotherapy or other immunosuppressive measures | x | x |
|  | J02.0 | Streptococcal pharyngitis | x | x |
|  | J02.8 | Acute pharyngitis due to other specified organisms | x | x |
|  | J03.0 | Streptococcal tonsillitis | x | x |
|  | J03.8 | Acute tonsillitis due to other specified organisms | x | x |
|  | J13 | Pneumonia due to Streptococcus pneumoniae | x | x |
|  | J15 | Bacterial pneumonia, not elsewhere classified | x | x |
|  | J16.0 | Chlamydial pneumonia | x | x |
|  | J17.0 | Pneumonia in bacterial diseases classified elsewhere | x | x |
|  | J20.0 | Acute bronchitis due to Mycoplasma pneumoniae | x | x |
|  | J20.1 | Acute bronchitis due to Haemophilus influenzae | x | x |
|  | J20.2 | Acute bronchitis due to streptococcus | x | x |
|  | J20.8 | Acute bronchitis due to other specified organisms | x | x |
|  | J21.8 | Acute bronchiolitis due to other specified organisms | x | x |
|  | J41 | Simple and mucopurulent chronic bronchitis | x | x |
|  | J42 | Unspecified chronic bronchitis | x | x |
|  | J44 | Other chronic obstructive pulmonary disease | x | x |
|  | ***ATC*** |  |  |  |
|  | J01AA02 | Doxycycline |  | x |
|  | J01CA04 | Amoxicillin |  | x |
|  | J01CE02 | Phenoxymethylpenicillin |  | x |
|  | J01CR02 | Amoxicillin and beta-lactamase inhibitor |  | x |
|  | J01FA01 | Erythromycin |  | x |
|  | J01FA09 | Clarithromycin |  | x |
| **Antipsychotics as first choice for dementia** | ***ICD*** |  |  |  |
|  | F20 – F29 | Schizophrenia, schizotypal and delusional disorders | x | x |
|  | F30 | Manic episode | x | x |
|  | F31 | Bipolar affective disorder | x | x |
| **Benzodiazepines as first choice for older persons** | ***ICD*** |  |  |  |
|  | F40 | Phobic anxiety disorders |  | x |
|  | F41 | Other anxiety disorders |  | x |
|  | G40 | Epilepsy |  | x |
| **Cough and cold medications** | No excluding services or diagnoses. | | | |
| **Ineffective drugs (such as selected nootropics) for Alzheimer disease** | No excluding services or diagnoses. | | | |
| **Opioids for acute non-specific back pain** | ***ICD*** |  |  |  |
|  | C00 – C97 | Malignant neoplasms | x | x |
|  | D37 – D48 | Neoplasms of uncertain or unknown behaviour | x | x |
|  | F11.2 | Mental and behavioural disorders due to use of opioids: Dependence syndrome | x | x |
|  | M00 – M25 | Arthropathies | x | x |
|  | M30 – M36 | Systemic connective tissue disorders | x | x |
|  | M40 – M43 | Deforming dorsopathies | x | x |
|  | M45 – M49 | Spondylopathies | x | x |
|  | M50 | Cervical disc disorders | x | x |
|  | M51 | Other intervertebral disc disorders | x | x |
|  | M53 | Other dorsopathies, not elsewhere classified | x | x |
|  | M60 – M79 | Soft tissue disorders | x | x |
|  | M80 – M94 | Osteopathies and chondropathies | x | x |
|  | M95 – M99 | Other disorders of the musculoskeletal system and connective tissue | x | x |
|  | Q35 – Q37 | Cleft lip and cleft palate | x | x |
|  | Q65 – Q79 | Congenital malformations and deformations of the musculoskeletal system | x | x |
|  | Q80 – Q89 | Congenital malformations and deformations of the musculoskeletal system | x | x |
|  | S00 – S09 | Injuries to the head | x | x |
|  | S10 – S19 | Injuries to the neck | x | x |
|  | S20 – S29 | Injuries to the thorax | x | x |
|  | S30 – S39 | Injuries to the abdomen, lower back, lumbar spine and pelvis | x | x |
|  | S40 – S49 | Injuries to the shoulder and upper arm | x | x |
|  | S50 – S59 | Injuries to the elbow and forearm | x | x |
|  | S60 – S69 | Injuries to the wrist and hand | x | x |
|  | S70 – S79 | Injuries to the hip and thigh | x | x |
|  | S80 – S89 | Injuries to the knee and lower leg | x | x |
|  | S90 – S99 | Injuries to the ankle and foot | x | x |
|  | T00 – T07 | Injuries involving multiple body regions | x | x |
|  | T08 – T14 | Injuries to unspecified part of trunk, limb or body region | x | x |
|  | T15 – T19 | Effects of foreign body entering through natural orifice | x | x |
|  | T20 – T32 | Burns and corrosions | x | x |
|  | T33 – T35 | Frostbite | x | x |
|  | T66 – T78 | Other and unspecified effects of external causes | x | x |
|  | T79 | Certain early complications of trauma, not elsewhere classified | x | x |
|  | T80 – T88 | Complications of surgical and medical care, not elsewhere classified | x | x |
|  | T89 | Other specified complications of trauma | x | x |
|  | T90 – T98 | Sequelae of injuries, of poisoning and o other consequences of external causes | x | x |
|  | V01 – X59 | Accidents | x | x |
|  | X60 – X84 | Intentional self-harm | x | x |
|  | X85 – Y09 | Assault | x | x |
|  | Y10 – Y34 | Event of undetermined intent | x | x |
|  | Y35 – Y36 | Legal intervention and operations of war | x | x |
|  | Y40 – Y84 | Complications of medical and surgical care | x | x |
| **Opioids for migraine or headache** | ***ICD*** |  |  |  |
|  | B20 – B24 | Human immunodeficiency virus [HIV] disease | x |  |
|  | C00 – C97 | Malignant neoplasms | x |  |
|  | D37 – D48 | Neoplasms of uncertain or unknown behaviour | x |  |
|  | F11.2 | Mental and behavioural disorders due to use of opioids: Dependence syndrome | x |  |
|  | G40 | Epilepsy | x |  |
|  | M00 – M99 | Diseases of the musculoskeletal system and connective tissue | x |  |
|  | Q35 – Q37 | Cleft lip and cleft palate | x |  |
|  | Q65 – Q79 | Congenital malformations and deformations of the musculoskeletal system | x |  |
|  | Q80 – Q89 | Congenital malformations and deformations of the musculoskeletal system | x |  |
|  | R10 | Abdominal and pelvic pain | x |  |
|  | R52.1 | Chronic intractable pain | x |  |
|  | S00 – S09 | Injuries to the head | x |  |
|  | S10 – S19 | Injuries to the neck | x |  |
|  | S20 – S29 | Injuries to the thorax | x |  |
|  | S30 – S39 | Injuries to the abdomen, lower back, lumbar spine and pelvis | x |  |
|  | S40 – S49 | Injuries to the shoulder and upper arm | x |  |
|  | S50 – S59 | Injuries to the elbow and forearm | x |  |
|  | S60 – S69 | Injuries to the wrist and hand | x |  |
|  | S70 – S79 | Injuries to the hip and thigh | x |  |
|  | S80 – S89 | Injuries to the knee and lower leg | x |  |
|  | S90 – S99 | Injuries to the ankle and foot | x |  |
|  | T00 – T07 | Injuries involving multiple body regions | x |  |
|  | T08 – T14 | Injuries to unspecified part of trunk, limb or body region | x |  |
|  | T15 – T19 | Effects of foreign body entering through natural orifice | x |  |
|  | T20 – T32 | Burns and corrosions | x |  |
|  | T33 – T35 | Frostbite | x |  |
|  | T66 – T78 | Other and unspecified effects of external causes | x |  |
|  | T79 | Certain early complications of trauma, not elsewhere classified | x |  |
|  | T80 – T88 | Complications of surgical and medical care, not elsewhere classified | x |  |
|  | T89 | Other specified complications of trauma | x |  |
|  | T90 – T98 | Sequelae of injuries, of poisoning and of other consequences of external causes | x |  |
|  | V01 – X59 | Accidents | x |  |
|  | X60 – X84 | Intentional self-harm | x |  |
|  | X85 – Y09 | Assault | x |  |
|  | Y10 – Y34 | Event of undetermined intent | x |  |
|  | Y35 – Y36 | Legal intervention and operations of war | x |  |
|  | Y40 – Y84 | Complications of medical and surgical care | x |  |
|  | Z32 | Pregnancy examination and test | x |  |
|  | Z33 | Pregnant state, incidental | x |  |
|  | Z34 | Supervision of normal pregnancy | x |  |
|  | Z35 | Supervision of high-risk pregnancy | x |  |
|  | Z64.0 | Problems related to unwanted pregnancy | x |  |
|  | ***OPS*** |  |  |  |
|  | 5-01 – 5-99 | Surgical procedures | x |  |
| **Diagnostic tests** | | | | |
| **Bone mineral density testing at frequent intervals** | ***ICD*** |  |  |  |
|  | S00 – S09 | Injuries to the head |  | x |
|  | S10 – S19 | Injuries to the neck |  | x |
|  | S20 – S29 | Injuries to the thorax |  | x |
|  | S30 – S39 | Injuries to the abdomen, lower back, lumbar spine and pelvis |  | x |
|  | S40 – S49 | Injuries to the shoulder and upper arm |  | x |
|  | S50 – S59 | Injuries to the elbow and forearm |  | x |
|  | S60 – S69 | Injuries to the wrist and hand |  | x |
|  | S70 – S79 | Injuries to the hip and thigh |  | x |
|  | S80 – S89 | Injuries to the knee and lower leg |  | x |
|  | S90 – S99 | Injuries to the ankle and foot |  | x |
|  | T00 – T07 | Injuries involving multiple body regions |  | x |
|  | T08 – T14 | Injuries to unspecified part of trunk, limb or body region |  | x |
|  | ***ATC*** |  |  |  |
|  | H02AB | Glucocorticoids |  | x |
|  | R03BA | Glucocorticoids |  | x |
| **Colonoscopy for constipation** | ***ICD*** |  |  |  |
|  | D50 – D53 | Nutritional anaemias | x | x |
|  | D55 – D59 | Haemolytic anaemias | x | x |
|  | D60 – D64 | Aplastic and other anaemias | x | x |
|  | D65 – D69 | Coagulation defects, purpura and other haemorrhagic conditions | x | x |
|  | R63.4 | Abnormal weight loss | x | x |
|  | Z80.0 | Family history of malignant neoplasm of digestive organs | x | x |
|  | Z85.0 | Personal history of malignant neoplasm of digestive organs | x | x |
|  | Z87.1 | Personal history of diseases of the digestive system | x | x |
| **EEG for headache** | ***ICD*** |  |  |  |
|  | F10.3 | Mental and behavioural disorders due to use of alcohol: Withdrawal state | x | x |
|  | F00 – F99 | Mental and behavioural disorders |  | x |
|  | G40 | Epilepsy | x | x |
|  | G43.3 | Complicated migraine | x | x |
|  | G00 – G99 | Diseases of the nervous system |  | x |
|  | R55 | Syncope and collapse | x | x |
|  | R56 | Convulsions, not elsewhere classified | x | x |
| **Endometrial biopsy for evaluation of infertility** | ***ICD*** |  |  |  |
|  | C00 – C97 | Malignant neoplasms | x | x |
| **Imaging for acute non-specific back pain** | ***ICD*** |  |  |  |
|  | C00 – C97 | Malignant neoplasms | x | x |
|  | D37 – D48 | Neoplasms of uncertain or unknown behaviour | x | x |
|  | M00 – M25 | Arthropathies | x | x |
|  | M30 – M36 | Systemic connective tissue disorders | x | x |
|  | M40 – M43 | Deforming dorsopathies | x | x |
|  | M45 – M49 | Spondylopathies | x | x |
|  | M50 | Cervical disc disorders | x | x |
|  | M51 | Other intervertebral disc disorders | x | x |
|  | M53 | Other dorsopathies, not elsewhere classified | x | x |
|  | M60 – M79 | Soft tissue disorders | x | x |
|  | M80 – M94 | Osteopathies and chondropathies | x | x |
|  | M95 – M99 | Other disorders of the musculoskeletal system and connective tissue | x | x |
|  | Q35 – Q37 | Cleft lip and cleft palate | x | x |
|  | Q65 – Q79 | Congenital malformations and deformations of the musculoskeletal system | x | x |
|  | Q80 – Q89 | Other concenital malformations | x | x |
|  | S00 – S09 | Injuries to the head | x | x |
|  | S10 – S19 | Injuries to the neck | x | x |
|  | S20 – S29 | Injuries to the thorax | x | x |
|  | S30 – S39 | Injuries to the abdomen, lower back, lumbar spine and pelvis | x | x |
|  | S40 – S49 | Injuries to the shoulder and upper arm | x | x |
|  | S50 – S59 | Injuries to the elbow and forearm | x | x |
|  | S60 – S69 | Injuries to the wrist and hand | x | x |
|  | S70 – S79 | Injuries to the hip and thigh | x | x |
|  | S80 – S89 | Injuries to the knee and lower leg | x | x |
|  | S90 – S99 | Injuries to the ankle and foot | x | x |
|  | T00 – T07 | Injuries involving multiple body regions | x | x |
|  | T08 – T14 | Injuries to unspecified part of trunk, limb or body region | x | x |
|  | T15 – T19 | Effects of foreign body entering through natural orifice | x | x |
|  | T20 – T32 | Burns and corrosions | x | x |
|  | T33 – T35 | Frostbite | x | x |
|  | T66 – T78 | Other and unspecified effects of external causes | x | x |
|  | T79 | Certain early complications of trauma, not elsewhere classified | x | x |
|  | T80 – T88 | Complications of surgical and medical care, not elsewhere classified | x | x |
|  | T89 | Other specified complications of trauma | x | x |
|  | T90 – T98 | Sequelae of injuries, of poisoning and of other consequences of external causes | x | x |
|  | V01 – X59 | Accidents | x | x |
|  | X60 – X84 | Intentional self-harm | x | x |
|  | X85 – Y09 | Assault | x | x |
|  | Y10 – Y34 | Event of undetermined intent | x | x |
|  | Y35 – Y36 | Legal intervention and operations of war | x | x |
|  | Y40 – Y84 | Complications of medical and surgical care | x | x |
| **Gastroscopy for dyspepsia** | ***ICD*** |  |  |  |
|  | D50 – D53 | Nutritional anaemias | x | x |
|  | D55 – D59 | Haemolytic anaemias | x | x |
|  | D60 – D64 | Aplastic and other anaemias | x | x |
|  | D65 – D69 | Coagulation defects, purpura and other haemorrhagic conditions | x | x |
|  | K21 | Gastro-oesophageal reflux disease |  | x |
|  | K22 | Perforation of oesophagus |  | x |
|  | K23 | Disorders of oesophagus in disease classified elsewhere |  | x |
|  | K25 | Gastric ulcer |  | x |
|  | K26 | Duodenal ulcer |  | x |
|  | K27 | Peptic ulcer, site unspecified |  | x |
|  | K28 | Gastrojejunal ulcer |  | x |
|  | K29 | Gastritis and duodenitis |  | x |
|  | K31 | Other diseases of stomach and duodenum |  | x |
|  | R13 | Dysphagia | x | x |
|  | R63.4 | Abnormal weight loss | x | x |
|  | Z80.0 | Family history of malignant neoplasm of digestive organs |  | x |
|  | Z85.0 | Personal history of malignant neoplasm of digestive organs |  | x |
|  | Z87.1 | Personal history of diseases of the digestive system |  | x |
| **Imaging for migraine or headache** | ***ICD*** |  |  |  |
|  | C69 – C72 | Malignant neoplasms of eye, brain and other parts of central nervous system | x | x |
|  | D80 - D89 | Certain disorders involving the immune mechanism | x | x |
|  | D90 | Immune compromise due to radiation, chemotherapy or other immunosuppressive measures | x | x |
|  | G40 | Epilepsy | x | x |
|  | G43.2 | Status migrainosus | x | x |
|  | G43.3 | Complicated migraine |  | x |
|  | G43.8 | Other migraine |  | x |
|  | G43.9 | Migraine, unspecified |  | x |
|  | G44.0 | Cluster headache syndrome | x | x |
|  | G44.1 | Vascular headache, not elsewhere classified | x | x |
|  | G44.3 | Chronic post-traumatic headache | x | x |
|  | G44.4 | Drug-induced headache, not elsewhere classified | x | x |
|  | G44.8 | Other specified headache syndromes | x | x |
|  | H47 | Other disorders of optic [2nd] nerve and visual pathways | x | x |
|  | R29.1 | Meningismus | x | x |
|  | R29.2 | Abnormal reflex | x | x |
|  | R29.3 | Abnormal posture | x | x |
|  | R29.5 | Neurological neglect syndrome | x | x |
|  | R29.6 | Tendency to fall, not elsewhere classified | x | x |
|  | R29.8 | Other and unspecified symptoms and signs involving the nervous and musculoskeletal systems | x | x |
|  | R50 | Fever of other and unknown origin | x | x |
|  | S02 | Fracture of skull and facial bones | x | x |
|  | S04 | Injury of cranial nerves | x | x |
|  | S06 | Intracranial injury | x | x |
|  | S07 | Crushing injury of head | x | x |
|  | S08 | Traumatic amputation of part of head | x | x |
|  | S09 | Other and unspecified injuries of head | x | x |
| **Preoperative chest radiography prior to selected surgeries** | ***ICD*** |  |  |  |
|  | C00 – C97 | Malignant neoplasms | x | x |
|  | E01 | Iodine-deficiency related thyroid disorders and allied conditions | x | x |
|  | E04 | Other nontoxic goitre | x | x |
|  | E05 | Thyrotoxicosis [hyperthyroidism] | x | x |
|  | I00 – I99 | Diseases of the circulatory system |  | x |
|  | J95 – J99 | Other diseases of the respiratory system |  | x |
|  | M40 – M43 | Deforming dorsopathies | x | x |
|  | ***EBM*** |  |  |  |
|  | 31181 | Cardiac surgery procedure with an incision-to-closure time up to 15 minutes | x | x |
|  | 31182 | Cardiac surgery procedure with an incision-to-closure time between 15 and 30 minutes | x | x |
|  | 31183 | Cardiac surgery procedure with an incision-to-closure time between 30 and 45 minutes | x | x |
|  | 31184 | Cardiac surgery procedure with an incision-to-closure time between 45 and 60 minutes | x | x |
|  | 31185 | Cardiac surgery procedure with an incision-to-closure time between 60 and 90 minutes | x | x |
|  | 31186 | Cardiac surgery procedure with an incision-to-closure time between 90 and 120 minutes | x | x |
|  | 31187 | Cardiac surgery procedure with an incision-to-closure time above 120 minutes | x | x |
|  | 31191 | Thoracic surgical procedure with an incision-to-closure time up to 15 minutes | x | x |
|  | 31192 | Thoracic surgical procedure with an incision-to-closure time between 15 and 30 minutes | x | x |
|  | 31193 | Thoracic surgical procedure with an incision-to-closure time between 30 and 45 minutes | x | x |
|  | 31194 | Thoracic surgical procedure with an incision-to-closure time between 45 and 60 minutes | x | x |
|  | 31195 | Thoracic surgical procedure with an incision-to-closure time between 60 and 90 minutes | x | x |
|  | 31196 | Thoracic surgical procedure with an incision-to-closure time between 90 and 120 minutes | x | x |
|  | 31197 | Thoracic surgical procedure with an incision-to-closure time above 120 minutes | x | x |
|  | ***OPS*** |  |  |  |
|  | 5-32 | Excision and resection in lung and bronchus | x | x |
|  | 5-33 | Other operations on lungs and bronchus | x | x |
|  | 5-34 | Operations on chest wall, pleura, mediastinum and diaphragm | x | x |
|  | 5-35 | Operations on cardiac valves and septa and vessels near the heart | x | x |
|  | 5-36 | Operations on the coronary vessels | x | x |
|  | 5-37 | Rhythm surgery and other operations on heart and pericardium | x | x |
| **Preoperative stress testing prior to selected surgeries** | ***ICD*** |  |  |  |
|  | I05 – I09 | Chronic rheumatic heart disease | x | x |
|  | I10 – I15 | Hypertensive diseases | x | x |
|  | I20 – I25 | Ischaemic heart diseases | x | x |
|  | I26 – I28 | Pulmonary heart disease and diseases of pulmonary circulation | x | x |
|  | I30 – I52 | Other forms of heart disease | x | x |
|  | R00 | Abnormalities of heart beat | x | x |
|  | ***EBM*** |  |  |  |
|  | 31181 | Cardiac surgery procedure with an incision-to-closure time up to 15 minutes | x | x |
|  | 31182 | Cardiac surgery procedure with an incision-to-closure time between 15 and 30 minutes | x | x |
|  | 31183 | Cardiac surgery procedure with an incision-to-closure time between 30 and 45 minutes | x | x |
|  | 31184 | Cardiac surgery procedure with an incision-to-closure time between 45 and 60 minutes | x | x |
|  | 31185 | Cardiac surgery procedure with an incision-to-closure time between 60 and 90 minutes | x | x |
|  | 31186 | Cardiac surgery procedure with an incision-to-closure time between 90 and 120 minutes | x | x |
|  | 31187 | Cardiac surgery procedure with an incision-to-closure time above 120 minutes | x | x |
|  | 31191 | Thoracic surgical procedure with an incision-to-closure time up to 15 minutes | x | x |
|  | 31192 | Thoracic surgical procedure with an incision-to-closure time between 15 and 30 minutes | x | x |
|  | 31193 | Thoracic surgical procedure with an incision-to-closure time between 30 and 45 minutes | x | x |
|  | 31194 | Thoracic surgical procedure with an incision-to-closure time between 45 and 60 minutes | x | x |
|  | 31195 | Thoracic surgical procedure with an incision-to-closure time between 60 and 90 minutes | x | x |
|  | 31196 | Thoracic surgical procedure with an incision-to-closure time between 90 and 120 minutes | x | x |
|  | 31197 | Thoracic surgical procedure with an incision-to-closure time above 120 minutes | x | x |
|  | ***OPS*** |  |  |  |
|  | 5-32 | Excision and resection in lung and bronchus | x | x |
|  | 5-33 | Other operations on lungs and bronchus | x | x |
|  | 5-34 | Operations on chest wall, pleura, mediastinum and diaphragm | x | x |
|  | 5-35 | Operations on cardiac valves and septa and vessels near the heart | x | x |
|  | 5-36 | Operations on the coronary vessels | x | x |
|  | 5-37 | Rhythm surgery and other operations on heart and pericardium | x | x |
| **Stress echocardiography for detection of coronary artery disease in ACS** | No excluding services or diagnoses. | | | |
| **Stress testing for stable coronary disease** | No excluding services or diagnoses. | | | |
| **Spirometry for known COPD** | No excluding services or diagnoses. | | | |
| **Testing for group A streptococcal pharyngitis** | ***ICD*** |  |  |  |
|  | Z20 | Contact with and exposure to communicable diseases |  | x |
| **Free T3/T4 level testing for hypothyroidism** | ***ICD*** |  |  |  |
|  | C75.1 | Malignant neoplasm: Pituitary gland |  | x |
|  | D09.3 | Carcinoma in situ: Thyroid and other endocrine glands |  | x |
|  | D17 | Benign lipomatous neoplasm |  | x |
|  | D44.3 | Neoplasm of uncertain or unknown behaviour: Pituitary gland |  | x |
|  | E00 | Congenital iodine-deficiency syndrome |  | x |
|  | E23 | Hypofunction and other disorders of pituitary gland |  | x |
|  | E24.0 | Pituitary-dependent Cushing disease |  | x |
|  | E85 | Amyloidosis |  | x |
|  | E89 | Postprocedural endocrine and metabolic disorders, not elsewhere classified |  | x |
|  | G04 | Encephalitis, myelitis and encephalomyelitis |  | x |
|  | M14.5 | Arthropathies in other endocrine, nutritional and metabolic disorders |  | x |
|  | S06 | Intracranial injury |  | x |
| **Tumour marker testing without cancer diagnosis** | ***ICD*** |  |  |  |
|  | B18.2 | Chronic viral hepatitis C |  | x |
|  | C00 – C97 | Malignant neoplasms | x | x |
|  | D00 – D09 | In situ neoplasms | x | x |
|  | K50 | Crohn disease [regional enteritis] |  | x |
|  | K51 | Ulcerative colitis |  | x |
|  | K74.3 | Primary biliary cirrhosis |  | x |
|  | K74.4 | Secondary biliary cirrhosis |  | x |
|  | K74.5 | Biliary cirrhosis, unspecified |  | x |
|  | K74.6 | Other and unspecified cirrhosis of liver |  | x |
|  | K86.1 | Other chronic pancreatitis |  | x |
|  | N83 | Noninflammatory disorders of ovary, fallopian tube and broad ligament |  | x |
|  | U55 | On waiting list for organ transplant |  | x |
|  | Z75.6 | Successful registration for organ transplantation without high urgency status |  | x |
|  | Z75.7 | Successful registration for organ transplantation with high urgency status |  | x |
|  | Z94 | Transplanted organ and tissue status |  | x |
| **Screening** | | | | |
| **Cancer screening for dialysis-dependent chronic kidney disease** | No excluding services or diagnoses. | | | |
| **Colorectal cancer screening for older persons** | ***ICD*** |  |  |  |
|  | C18 | Malignant neoplasm of colon |  | x |
|  | K63.5 | Polyp of colon |  | x |
|  | Z80.0 | Family history of malignant neoplasm of digestive organs |  | x |
| **Mammography screening for older women** | ***ICD*** |  |  |  |
|  | C50 | Malignant neoplasm of breast |  | x |
|  | N62 | Hypertrophy of breast |  | x |
|  | N63 | Unspecified lump in breast |  | x |
| **Mammography screening for younger women** | ***ICD*** |  |  |  |
|  | C50 | Malignant neoplasm of breast |  | x |
|  | N60 – N64 | Disorders of breast |  | x |
|  | Z80 | Family history of primary malignant neoplasm |  | x |
|  | ***EBM*** |  |  |  |
|  | 07345 | Supplementary lump sum for treatment and/or care of a patient with a confirmed oncological disease during ongoing oncological therapy or follow-up care |  | x |
|  | 08345 | Supplementary lump sum for treatment and/or care of a patient with a confirmed oncological disease during ongoing oncological therapy or follow-up care |  | x |
|  | ***ATC*** |  |  |  |
|  | G03C | Sex hormones and modulators of the genital system: Estrogens |  | x |
|  | G03D | Sex hormones and modulators of the genital system: Progestogens |  | x |
|  | G03F | Sex hormones and modulators of the genital system: Progestogens and estrogens in combination |  | x |
| **Treatment** | | | | |
| **Abdominal hysterectomy for benign diseases** | ***ICD*** |  |  |  |
|  | C00 – C97 | Malignant neoplasms | x | x |
|  | N73.6 | Female pelvic peritoneal adhesions |  | x |
|  | N80 | Endometriosis |  | x |
|  | N99.4 | Postprocedural pelvic peritoneal adhesions |  | x |
|  | O82 | Single delivery by caesarean section | x | x |
|  | Z80 | Family history of primary malignant neoplasm | x | x |
|  | ***OPS*** |  |  |  |
|  | 5-74 | Caesarean section and child development | x | x |
| **Chemotherapy for cancer in the last months of life** | No excluding services or diagnoses. | | | |
| **Electrotherapy for pressure ulcer** | ***ICD*** |  |  |  |
|  | L98.4 | Chronic ulcer of skin, not elsewhere classified |  | x |
| **ERC for acute gallstone pancreatitis without cholangitis** | ***ICD*** |  |  |  |
|  | K80.3 | Calculus of bile duct with cholangitis | x | x |
|  | K80.41 | Calculus of bile duct with cholecystitis with obstruction of biliary tract | x | x |
|  | K80.51 | Calculus of bile duct without cholangitis or cholecystitis with obstruction of biliary tract | x | x |
|  | K83.0 | Cholangitis | x | x |
| **Epidural steroid injections for low back pain** | ***ICD*** |  |  |  |
|  | C00 – C97 | Malignant neoplasms | x | x |
|  | D37 – D48 | Neoplasms of uncertain or unknown behaviour | x | x |
|  | G55.1 | Nerve root and plexus compressions in intervertebral disc disorders | x | x |
|  | M00 – M25 | Arthropathies | x | x |
|  | M30 – M36 | Systemic connective tissue disorders | x | x |
|  | M40 – M43 | Dorsopathies | x | x |
|  | M45 – M49 | Spondylopathies | x | x |
|  | M50 | Cervical disc disorders | x | x |
|  | M51 | Other intervertebral disc disorders | x | x |
|  | M53 | Other dorsopathies, not elsewhere classified | x | x |
|  | M54.15 | Radiculopathy thoracolumbar region | x | x |
|  | M54.16 | Radiculopathy lumbar region | x | x |
|  | M54.17 | Radiculopathy lumbosacral region | x | x |
|  | M54.4 | Lumbago with sciatica | x | x |
|  | M60 – M79 | Soft tissue disorders | x | x |
|  | M80 – M94 | Osteopathies and chondropathies | x | x |
|  | M95 – M99 | Other disorders of the musculoskeletal system and connective tissue | x | x |
|  | Q35 – Q37 | Cleft lip and cleft palate | x | x |
|  | Q65 – Q79 | Congenital malformations and deformations of the musculoskeletal system | x | x |
|  | Q80 – Q89 | Other congenital malformations | x | x |
|  | S00 – S09 | Injuries to the head | x | x |
|  | S10 – S19 | Injuries to the neck | x | x |
|  | S20 – S29 | Injuries to the thorax | x | x |
|  | S30 – S39 | Injuries to the abdomen, lower back, lumbar spine and pelvis | x | x |
|  | S40 – S49 | Injuries to the shoulder and upper arm | x | x |
|  | S50 – S59 | Injuries to the elbow and forearm | x | x |
|  | S60 – S69 | Injuries to the wrist and hand | x | x |
|  | S70 – S79 | Injuries to the hip and thigh | x | x |
|  | S80 – S89 | Injuries to the knee and lower leg | x | x |
|  | S90 – S99 | Injuries to the ankle and foot | x | x |
|  | T00 – T07 | Injuries involving multiple body regions | x | x |
|  | T08 – T14 | Injuries to unspecified part of trunk, limb or body region | x | x |
|  | T15 – T19 | Effects of foreign body entering through natural orifice | x | x |
|  | T20 – T32 | Burns and corrosions | x | x |
|  | T33 – T35 | Frostbite | x | x |
|  | T66 – T78 | Other and unspecified effects of external causes | x | x |
|  | T79 | Certain early complications of trauma, not elsewhere classified | x | x |
|  | T80 – T88 | Complications of surgical and medical care, not elsewhere classified | x | x |
|  | T89 | Other specified complications of trauma | x | x |
|  | T90 – T98 | Sequelae of injuries, of poisoning and of other consequences of external causes | x | x |
|  | V01 – X59 | Accidents | x | x |
|  | X60 – X84 | Intentional self-harm | x | x |
|  | X85 – Y09 | Assault | x | x |
|  | Y10 – Y34 | Event of undetermined intent | x | x |
|  | Y35 – Y36 | Legal intervention and operations of war | x | x |
|  | Y40 – Y84 | Complications of medical and surgical care | x | x |
| **Inhalation therapy for COPD without previously confirming the diagnosis by spirometry** | ***EBM*** |  |  |  |
|  | 03330 | Spirographic examination | x | x |
|  | 27330 | Spirographic examination | x | x |
|  | 13255 | Spirographic examination | x | x |
| **PTA of the renal artery or stenting for selected diagnoses** | ***ICD*** |  |  |  |
|  | I77.3 | Arterial fibromuscular dysplasia | x | x |
|  | J81 | Pulmonary oedema | x | x |
| **Postoperative radiation therapy after radical prostatectomy** | No excluding services or diagnoses. | | | |
| **Removal of gallbladder during bariatric surgery** | ***ICD*** |  |  |  |
|  | C23 | Malignant neoplasm of gallbladder | x |  |
|  | D01.5 | Carcinoma in situ: Liver, gallbladder and bile ducts | x |  |
|  | D37.6 | Neoplasm of uncertain or unknown behaviour: Liver, gallbladder and bile ducts | x |  |
|  | K80.00 | Calculus of gallbladder with acute cholecystitis: Without mention of obstruction of biliary tract | x |  |
|  | K80.01 | Calculus of gallbladder with acute cholecystitis: With obstruction of biliary tract | x |  |
|  | K80.10 | Calculus of gallbladder with other cholecystitis: Without mention of obstruction of biliary tract | x |  |
|  | K80.11 | Calculus of gallbladder with other cholecystitis: With obstruction of biliary tract | x |  |
|  | K80.20 | Calculus of gallbladder without cholecystitis: Without mention of obstruction of biliary tract | x |  |
|  | K80.21 | Calculus of gallbladder without cholecystitis: With obstruction of biliary tract | x |  |
|  | K82 | Other diseases of gallbladder | x |  |
|  | K87 | Disorders of gallbladder, biliary tract and pancreas in diseases classified elsewhere | x |  |
|  | Q44.0 | Agenesis, aplasia and hypoplasia of gallbladder | x |  |
|  | Q44.1 | Other congenital malformations of gallbladder | x |  |
| **Retinal laser therapy or cryotherapy for asymptomatic lattice degeneration** | ***ICD*** |  |  |  |
|  | H33 | Retinal detachments and breaks | x | x |
| **Spinal fusion for low back pain** | ***ICD*** |  |  |  |
|  | G55.1 | Nerve root and plexus compressions in intervertebral disc disorders | x | x |
|  | M40.00 | Postural kyphosis: Multiple sites in spine | x | x |
|  | M40.06 | Postural kyphosis: Lumbar region | x | x |
|  | M40.07 | Postural kyphosis: Lumbosacral region | x | x |
|  | M40.08 | Postural kyphosis: Sacral and sacrococcygeal region | x | x |
|  | M40.09 | Postural kyphosis: Site unspecified | x | x |
|  | M40.10 | Other secondary kyphosis: Multiple sites in spine | x | x |
|  | M40.16 | Other secondary kyphosis: Lumbar region | x | x |
|  | M40.17 | Other secondary kyphosis: Lumbosacral region | x | x |
|  | M40.18 | Other secondary kyphosis: Sacral and sacrococcygeal region | x | x |
|  | M40.19 | Other secondary kyphosis: Site unspecified | x | x |
|  | M40.30 | Flatback syndrome: Multiple sites in spine | x | x |
|  | M40.36 | Flatback syndrome: Lumbar region | x | x |
|  | M40.37 | Flatback syndrome: Lumbosacral region | x | x |
|  | M40.38 | Flatback syndrome: Sacral and sacrococcygeal region | x | x |
|  | M40.39 | Flatback syndrome: Site unspecified | x | x |
|  | M40.40 | Other lordosis: Multiple sites in spine | x | x |
|  | M40.46 | Other lordosis: Lumbar region | x | x |
|  | M40.47 | Other lordosis: Lumbosacral region | x | x |
|  | M40.48 | Other lordosis: Sacral and sacrococcygeal region | x | x |
|  | M40.49 | Other lordosis: Multiple sites in spine | x | x |
|  | M41 | Scoliosis | x | x |
|  | M42 | Spinal osteochondrosis | x | x |
|  | M43.1 | Spondylolisthesis | x | x |
|  | M43.20 | Other fusion of spine: Multiple sites in spine | x | x |
|  | M43.26 | Other fusion of spine: Lumbar region | x | x |
|  | M43.27 | Other fusion of spine: Lumbosacral region | x | x |
|  | M43.28 | Other fusion of spine: Sacral and sacrococcygeal region | x | x |
|  | M43.29 | Other fusion of spine: Site unspecified | x | x |
|  | M43.3 | Recurrent atlantoaxial subluxation with myelopathy | x | x |
|  | M43.4 | Other recurrent atlantoaxial subluxation | x | x |
|  | M43.50 | Other recurrent vertebral subluxation: Multiple sites in spine | x | x |
|  | M43.56 | Other recurrent vertebral subluxation: Lumbar region | x | x |
|  | M43.57 | Other recurrent vertebral subluxation: Lumbosacral region | x | x |
|  | M43.58 | Other recurrent vertebral subluxation: Sacral and sacrococcygeal region | x | x |
|  | M43.59 | Other recurrent vertebral subluxation: Site unspecified | x | x |
|  | M43.6 | Torticollis | x | x |
|  | M43.80 | Other specified deforming dorsopathies: Multiple sites in spine | x | x |
|  | M43.86 | Other specified deforming dorsopathies: Lumbar region | x | x |
|  | M43.87 | Other specified deforming dorsopathies: Lumbosacral region | x | x |
|  | M43.88 | Other specified deforming dorsopathies: Sacral and sacrococcygeal region | x | x |
|  | M43.89 | Other specified deforming dorsopathies: Site unspecified | x | x |
|  | M43.90 | Deforming dorsopathy, unspecified: Multiple sites in spine | x | x |
|  | M43.96 | Deforming dorsopathy, unspecified: Lumbar region | x | x |
|  | M43.97 | Deforming dorsopathy, unspecified: Lumbosacral region | x | x |
|  | M43.98 | Deforming dorsopathy, unspecified: Sacral and sacrococcygeal region | x | x |
|  | M43.99 | Deforming dorsopathy, unspecified: Site unspecified | x | x |
|  | M51.1 | Lumbar and other intervertebral disc disorders with radiculopathy | x | x |
|  | M54.4 | Lumbago with sciatica | x | x |
|  | M79.65 | Pain in limb: Pelvic region and thigh | x | x |
|  | M79.66 | Pain in limb: Lower leg | x | x |
|  | M79.67 | Pain in limb: Ankle and foot | x | x |
|  | Q67.5 | Congenital deformity of spine | x | x |
|  | Q76.21 | Congenital spondylolisthesis | x | x |
|  | Q76.3 | Congenital scoliosis due to congenital bony malformation | x | x |
|  | Q76.4 | Other congenital malformations of spine, not associated with scoliosis | x | x |
| **Surgery for vesicoureteral reflux** | No excluding services or diagnoses. | | | |
| **Tube feeding via PEG in the last months of life** | No excluding services or diagnoses. | | | |
| **Unblocking nasolacrimal duct** | No excluding services or diagnoses. | | | |

**References**

1. Chua KP, Schwartz AL, Volerman A, Conti RM, Huang ES. Use of Low-Value Pediatric Services Among the Commercially Insured. Pediatrics. 2016;138:e20161809. doi:10.1542/peds.2016-1809.

2. Chua KP, Schwartz AL, Volerman A, Conti RM, Huang ES. Differences in the Receipt of Low-Value Services Between Publicly and Privately Insured Children. Pediatrics. 2020;145:e20192325. doi:10.1542/peds.2019-2325.

3. Nowakowska M, van Staa T, Mölter A, Ashcroft DM, Tsang JY, White A, et al. Antibiotic choice in UK general practice: rates and drivers of potentially inappropriate antibiotic prescribing. J Antimicrob Chemother. 2019;74:3371–8. doi:10.1093/jac/dkz345.

4. Brett J, Zoega H, Buckley NA, Daniels BJ, Elshaug AG, Pearson SA. Choosing wisely? Quantifying the extent of three low value psychotropic prescribing practices in Australia. BMC Health Serv Res. 2018;18:1009. doi:10.1186/s12913-018-3811-5.

5. Colla CH, Morden NE, Sequist TD, Schpero WL, Rosenthal MB. Choosing wisely: prevalence and correlates of low-value health care services in the United States. J Gen Intern Med. 2015;30:221–8. doi:10.1007/s11606-014-3070-z.

6. Puyat JH, Law MR, Wong ST, Sutherland JM, Morgan SG. The essential and potentially inappropriate use of antipsychotics across income groups: an analysis of linked administrative data. Can J Psychiatry. 2012;57:488–95. doi:10.1177/070674371205700807.

7. Singer A, Kosowan L, Katz A, Jolin-Dahel K, Appel K, Lix LM. Prescribing and testing by primary care providers to assess adherence to the Choosing Wisely Canada recommendations: a retrospective cohort study. CMAJ Open. 2018;6:E603-E610. doi:10.9778/cmajo.20180053.

8. Schubert I, Siegel A, Köster I, Ihle P. Evaluation der populationsbezogenen ‚Integrierten Versorgung Gesundes Kinzigtal‘ (IVGK). Ergebnisse zur Versorgungsqualität auf der Basis von Routinedaten. [Evaluation of the population-based 'Integrated Health Care System Gesundes Kinzigtal' (IHGK). Findings on health care quality based on administrative data]. Z Evid Fortbild Qual Gesundhwes. 2016;117:27–37. doi:10.1016/j.zefq.2016.06.003.

9. Barnett ML, Linder JA, Clark CR, Sommers BD. Low-Value Medical Services in the Safety-Net Population. JAMA Intern Med. 2017;177:829–37. doi:10.1001/jamainternmed.2017.0401.

10. Isaac T, Rosenthal MB, Colla CH, Morden NE, Mainor AJ, Li Z, et al. Measuring overuse with electronic health records data. Am J Manag Care. 2018;24:19–25.

11. Rosenthal MB, Colla CH, Morden NE, Sequist TD, Mainor AJ, Li Z, Nguyen KH. Overuse and insurance plan type in a privately insured population. Am J Manag Care. 2018;24:140–6.

12. Pozo-Rosich P, Layos-Romero A, Martin-Delgado J, Pascual J, Bailón C, Tentor A, et al. Low-value care practice in headache: a Spanish mixed methods research study. J Headache Pain. 2020;21:74. doi:10.1186/s10194-020-01147-w.

13. Schwartz AL, Landon BE, Elshaug AG, Chernew ME, McWilliams JM. Measuring low-value care in Medicare. JAMA Intern Med. 2014;174:1067–76. doi:10.1001/jamainternmed.2014.1541.

14. Schwartz AL, Jena AB, Zaslavsky AM, McWilliams JM. Analysis of Physician Variation in Provision of Low-Value Services. JAMA Intern Med. 2019;179:16–25. doi:10.1001/jamainternmed.2018.5086.

15. Chalmers K, Badgery-Parker T, Pearson SA, Brett J, Scott IA, Elshaug AG. Developing indicators for measuring low-value care: mapping Choosing Wisely recommendations to hospital data. BMC Res Notes. 2018;11:163. doi:10.1186/s13104-018-3270-4.

16. Badgery-Parker T, Pearson SA, Chalmers K, Brett J, Scott IA, Dunn S, et al. Low-value care in Australian public hospitals: prevalence and trends over time. BMJ Qual Saf. 2019;28:205–14. doi:10.1136/bmjqs-2018-008338.

17. Radomski TR, Feldman R, Huang Y, Sileanu FE, Thorpe CT, Thorpe JM, et al. Evaluation of Low-Value Diagnostic Testing for 4 Common Conditions in the Veterans Health Administration. JAMA Netw Open. 2020;3:e2016445. doi:10.1001/jamanetworkopen.2020.16445.

18. Linder R, Horenkamp-Sonntag D, Engel S, Schneider U, Verheyen F. Überdiagnostik mit Bildgebung bei Rückenschmerzen. [Quality Assurance using routine data: Overdiagnosis by radiological imaging for back pain]. Dtsch Med Wochenschr. 2016;141:e96-e103. doi:10.1055/s-0042-101467.

19. Rosenberg A, Agiro A, Gottlieb M, Barron J, Brady P, Liu Y, et al. Early Trends Among Seven Recommendations From the Choosing Wisely Campaign. JAMA Intern Med. 2015;175:1913–20. doi:10.1001/jamainternmed.2015.5441.

20. Bouck Z, Pendrith C, Chen XK, Frood J, Reason B, Khan T, et al. Measuring the frequency and variation of unnecessary care across Canada. BMC Health Serv Res. 2019;19:446. doi:10.1186/s12913-019-4277-9.

21. Carter EA, Morin PE, Lind KD. Costs and Trends in Utilization of Low-value Services Among Older Adults With Commercial Insurance or Medicare Advantage. Med Care. 2017;55:931–9. doi:10.1097/MLR.0000000000000809.

22. Romano MJ, Segal JB, Pollack CE. The Association Between Continuity of Care and the Overuse of Medical Procedures. JAMA Intern Med. 2015;175:1148–54. doi:10.1001/jamainternmed.2015.1340.

23. Koehlmoos TP, Madsen CK, Banaag A, Haider AH, Schoenfeld AJ, Weissman JS. Assessing Low-Value Health Care Services In The Military Health System. Health Aff (Millwood). 2019;38:1351–7. doi:10.1377/hlthaff.2019.00252.

24. Segal JB, Bridges JFP, Chang HY, Chang E, Nassery N, Weiner J, Chan KS. Identifying possible indicators of systematic overuse of health care procedures with claims data. Med Care. 2014;52:157–63. doi:10.1097/MLR.0000000000000052.

25. Segal JB, Nassery N, Chang HY, Chang E, Chan K, Bridges JFP. An index for measuring overuse of health care resources with Medicare claims. Med Care. 2015;53:230–6. doi:10.1097/MLR.0000000000000304.

26. Villar-Álvarez F, Moreno-Zabaleta R, Mira-Solves JJ, Calvo-Corbella E, Díaz-Lobato S, González-Torralba F, et al. Do not do in COPD: consensus statement on overuse. Int J Chron Obstruct Pulmon Dis. 2018;13:451–63. doi:10.2147/COPD.S151939.

27. Gion M, Cardinali G, Trevisiol C, Zappa M, Rainato G, Fabricio ASC. Indicators of inappropriate tumour marker use through the mining of electronic health records. J Eval Clin Pract. 2017;23:895–902. doi:10.1111/jep.12754.

28. Kale MS, Bishop TF, Federman AD, Keyhani S. Trends in the overuse of ambulatory health care services in the United States. JAMA Intern Med. 2013;173:142–8. doi:10.1001/2013.jamainternmed.1022.

29. Tran K, Rahal R, Fung S, Lockwood G, Louzado C, Xu J, Bryant H. Choosing wisely in cancer control across Canada-a set of baseline indicators. Curr Oncol. 2017;24:201–6. doi:10.3747/co.24.3643.

30. De Schreye R, Houttekier D, Deliens L, Cohen J. Developing indicators of appropriate and inappropriate end-of-life care in people with Alzheimer's disease, cancer or chronic obstructive pulmonary disease for population-level administrative databases: A RAND/UCLA appropriateness study. Palliat Med. 2017;31:932–45. doi:10.1177/0269216317705099.

31. De Schreye R, Smets T, Deliens L, Annemans L, Gielen B, Cohen J. Appropriateness of End-of-Life Care in People Dying With Dementia: Applying Quality Indicators on Linked Administrative Databases. J Am Med Dir Assoc. 2020;21:1093-1101.e1. doi:10.1016/j.jamda.2019.12.020.

32. Van Baal K, Schrader S, Schneider N, Wiese B, Stahmeyer JT, Eberhard S, et al. Quality indicators for the evaluation of end-of-life care in Germany - a retrospective cross-sectional analysis of statutory health insurance data. BMC Palliat Care. 2020;19:187. doi:10.1186/s12904-020-00679-x.

33. Radbruch L, Andersohn F, Walker J. Faktencheck Gesundheit – Palliativversorgung Modul 3: Überversorgung kurativ – Unterversorgung palliativ? Analyse ausgewählter Behandlungen am Lebensende. 2015.
