# Supplementary material for: Selecting indicators for the measurement of low-value care using German claims data: A three-round modified Delphi panel
Source: PLoS One. 2025 Feb 18;20(2):e0314864. doi: 10.1371/journal.pone.0314864 (PMC11835324; doi:10.1371/journal.pone.0314864)
Supplement: S3 Table — Abbreviations: ACS = Acute Coronary Syndrome; COPD = Chronic Obstructive Pulmonary Disease; EEG = Electroencephalography; ERC = Endoscopic Retrograde Cholangiography; PEG = Percutaneous Endoscopic Gastrostomy; PTA = Percutaneous Transluminal Angioplasty. (DOCX) [file pone.0314864.s003.docx]

**S6: Number of respondents per indicator.**

| **Indicator** | **Number of respondents** | | |
| --- | --- | --- | --- |
|  | **Round 1** | **Round 3** |  |
| **Pharmaceuticals** |  |  |  |
| **Acid blockers for uncomplicated gastroesophageal reflux** | 2 | 2 |  |
| **Antibiotics for acute otitis media** | 3 | 3 |  |
| **Antibiotics for uncomplicated respiratory tract infections** | 9 | 8 |  |
| **Antipsychotics as first choice for dementia** | 5 | 4 |  |
| **Benzodiazepines as first choice for older persons** | 6 | 4 |  |
| **Cough and cold medications** | 3 | 3 |  |
| **Ineffective drugs (such as selected nootropics) for Alzheimer disease** | 5 | 4 |  |
| **Opioids for acute non-specific back pain** | 7 | 7 |  |
| **Opioids for migraine or headache** | 5 | 5 |  |
| **Diagnostic tests** |  |  |  |
| **Bone mineral density testing at frequent intervals** | 5 | 5 |  |
| **Colonoscopy for constipation** | 10 | 8 |  |
| **EEG for headache** | 5 | 5 |  |
| **Endometrial biopsy for investigation of infertility** | 5 | 4 |  |
| **Gastroscopy for dyspepsia** | 3 | 2 |  |
| **Imaging for acute non-specific back pain** | 6 | 6 |  |
| **Imaging for migraine or headache** | 5 | 5 |  |
| **Preoperative chest radiography prior to selected surgeries** | 13 | 13 |  |
| **Preoperative stress testing prior to selected surgeries** | 12 | 12 |  |
| **Stress echocardiography for detection of coronary artery disease in ACS** | 7 | 6 |  |
| **Stress testing for stable coronary disease** | 6 | 5 |  |
| **Spirometry for known COPD** | 5 | 4 |  |
| **Testing for group A streptococcal pharyngitis** | 3 | 3 |  |
| **Free T3/T4 level testing for hypothyroidism** | 4 | 3 |  |
| **Tumour marker testing without cancer diagnosis** | 8 | 8 |  |
| **Screening** |  |  |  |
| **Cancer screening for dialysis-dependent chronic kidney disease** | 8 | 7 |  |
| **Colorectal cancer screening for older persons** | 10 | 8 |  |
| **Mammography screening in older women** | 9 | - |  |
| **Mammography screening in younger women** | 8 | - |  |
| **Treatment** |  |  |  |
| **Abdominal hysterectomy for benign diseases** | 6 | 5 |  |
| **Chemotherapy for cancer in the last months of life** | 10 | 7 |  |
| **Electrotherapy for pressure ulcer** | 6 | 4 |  |
| **ERC for calculus of bile duct or acute pancreatitis without cholangitis** | 7 | 6 |  |
| **Epidural steroid injections for low back pain** | 5 | 5 |  |
| **Inhalation therapy for COPD without previously confirming the diagnosis by spirometry** | 5 | 4 |  |
| **PTA of the renal artery or stenting for selected diagnoses** | 5 | 4 |  |
| **Postoperative radiation therapy after radical prostatectomy** | 4 | 4 |  |
| **Removal of gallbladder during bariatric surgery** | 7 | 6 |  |
| **Retinal laser therapy or cryotherapy for asymptomatic lattice degeneration** | 2 | - |  |
| **Spinal fusion for low back pain** | 4 | 4 |  |
| **Surgery for vesicoureteral reflux** | 2 | 2 |  |
| **Tube feeding via PEG for dementia in the last months of life** | 9 | 7 |  |
| **Unblocking nasolacrimal duct** | 3 | 3 |  |
