# Supplementary material for: Selecting indicators for the measurement of low-value care using German claims data: A three-round modified Delphi panel
Source: PLoS One. 2025 Feb 18;20(2):e0314864. doi: 10.1371/journal.pone.0314864 (PMC11835324; doi:10.1371/journal.pone.0314864)
Supplement: S4 Table — (DOCX) [file pone.0314864.s004.docx]

# **S7: Excluding diagnoses and services**

| **Indicator** | **Excluding services and diagnoses** | | **Sensitive** | **Specific** |
| --- | --- | --- | --- | --- |
| **Pharmaceuticals** | | | | |
| **Acid blockers for uncomplicated gastroesophageal reflux** | ***ICD*** |  |  |  |
|  | K20 | Oesophagitis |  | x |
|  | K25 | Gastric ulcer |  | x |
|  | K26 | Duodenal ulcer |  | x |
|  | K27 | Peptic ulcer, site unspecified |  | x |
|  | K28 | Gastrojejunal ulcer |  | x |
|  | K29 | Gastritis and duodenitis |  | x |
|  | K92.0 | Haematemesis |  | x |
|  | K92.8 | Other specified diseases of digestive system |  | x |
|  | P05.0 | Light for gestational age |  | x |
|  | P28.3 | Primary sleep apnea of newborn |  | x |
|  | P28.4 | Other apnea of newborn |  | x |
|  | P54.3 | Other neonatal gastrointestinal haemorrhage |  | x |
|  | P92.3 | Underfeeding of newborn |  | x |
|  | R06.8 | Other and unspecified abnormalities of breathing |  | x |
|  | R45.4 | Irritability and anger |  | x |
|  | R62 | Lack of expected normal physiological development |  | x |
|  | R63.4 | Abnormal weight loss |  | x |
|  | R68.1 | Nonspecific symptoms peculiar to infancy |  | x |
| **Antibiotics for acute otitis media** | ***ICD*** |  |  |  |
|  | A49 | Bacterial infection of unspecified site | x | x |
|  | A15 – A19 | Tuberculosis | x | x |
|  | A20 – A28 | Certain zoonotic bacterial diseases | x | x |
|  | B90.8 | Sequelae of tuberculosis of other organs | x | x |
|  | B90.9 | Sequelae of respiratory and unspecified tuberculosis | x | x |
|  | D80 – D89 | Certain disorders involving the immune mechanism | x | x |
|  | D90 | Immune comprise due to radiation, chemotherapy or other immunosuppressive measures | x | x |
|  | J02.0 | Streptococcal pharyngitis | x | x |
|  | J03.0 | Streptococcal tonsillitis | x | x |
|  | J13 | Pneumonia due to Streptococcus pneumoniae | x | x |
|  | J14 | Pneumonia due to Haemophilus influenzae | x | x |
|  | J15 | Bacterial pneumonia, not elsewhere classified | x | x |
|  | J16.0 | Chlamydial pneumonia | x | x |
|  | J17.0 | Pneumonia in bacterial diseases classified elsewhere | x | x |
|  | J20.0 | Acute bronchitis due to Mycoplasma pneumoniae | x | x |
|  | J20.1 | Acute bronchitis due to Haemophilus influenzae | x | x |
|  | J20.2 | Acute bronchitis due to streptococcus | x | x |
|  | J41 | Simple and mucopurulent chronic bronchitis | x | x |
|  | H60.0 | Abscess of external ear | x | x |
|  | H60.1 | Cellulitis of external ear | x | x |
|  | H60.3 | Other infective otitis externa | x | x |
|  | H60.4 | Cholesteatoma of external ear | x | x |
|  | H65.2 | Chronic serous otitis media | x | x |
|  | H65.3 | Chronic mucoid otitis media | x | x |
|  | H65.4 | Other chronic nonsuppurative otitis media | x | x |
|  | H66.0 | Acute suppurative otitis media | x | x |
|  | Q18 | Other congenital malformations of face and neck | x | x |
|  | Q87.0 | Congenital malformation syndromes predominantly affecting facial appearance | x | x |
|  | R50 | Fever of other and unknown origin | x | x |
|  | Z96.2 | Presence of otological and audiological implants | x | x |
|  | ***ATC*** |  |  |  |
|  | J01CA04 | Amoxicillin |  | x |
|  | J01FA | Macrolides |  | x |
| **Antibiotics for uncomplicated respiratory tract infections** | ***ICD*** |  |  |  |
|  | A49 | Bacterial infection of unspecified site | x | x |
|  | A15 – A19 | Tuberculosis | x | x |
|  | A20 – A28 | Certain zoonotic bacterial diseases | x | x |
|  | B90.8 | Sequelae of tuberculosis of other organs | x | x |
|  | B90.9 | Sequelae of respiratory and unspecified tuberculosis | x | x |
|  | D80 – D89 | Certain disorders involving the immune mechanism | x | x |
|  | D90 | Immune compromise due to radiation, chemotherapy or other immunosuppressive measures | x | x |
|  | J02.0 | Streptococcal pharyngitis | x | x |
|  | J02.8 | Acute pharyngitis due to other specified organisms | x | x |
|  | J03.0 | Streptococcal tonsillitis | x | x |
|  | J03.8 | Acute tonsillitis due to other specified organisms | x | x |
|  | J13 | Pneumonia due to Streptococcus pneumoniae | x | x |
|  | J15 | Bacterial pneumonia, not elsewhere classified | x | x |
|  | J16.0 | Chlamydial pneumonia | x | x |
|  | J17.0 | Pneumonia in bacterial diseases classified elsewhere | x | x |
|  | J20.0 | Acute bronchitis due to Mycoplasma pneumoniae | x | x |
|  | J20.1 | Acute bronchitis due to Haemophilus influenzae | x | x |
|  | J20.2 | Acute bronchitis due to streptococcus | x | x |
|  | J20.8 | Acute bronchitis due to other specified organisms | x | x |
|  | J21.8 | Acute bronchiolitis due to other specified organisms | x | x |
|  | J41 | Simple and mucopurulent chronic bronchitis | x | x |
|  | J42 | Unspecified chronic bronchitis | x | x |
|  | J44 | Other chronic obstructive pulmonary disease | x | x |
|  | ***ATC*** |  |  |  |
|  | J01AA02 | Doxycycline |  | x |
|  | J01CA04 | Amoxicillin |  | x |
|  | J01CE02 | Phenoxymethylpenicillin |  | x |
|  | J01CR02 | Amoxicillin and beta-lactamase inhibitor |  | x |
|  | J01FA01 | Erythromycin |  | x |
|  | J01FA09 | Clarithromycin |  | x |
| **Antipsychotics as first choice for dementia** | ***ICD*** |  |  |  |
|  | F20 – F29 | Schizophrenia, schizotypal and delusional disorders | x | x |
|  | F30 | Manic episode | x | x |
|  | F31 | Bipolar affective disorder | x | x |
| **Benzodiazepines as first choice for older persons** | ***ICD*** |  |  |  |
|  | F40 | Phobic anxiety disorders |  | x |
|  | F41 | Other anxiety disorders |  | x |
|  | G40 | Epilepsy |  | x |
| **Cough and cold medications** | No excluding services or diagnoses. | | | |
| **Ineffective drugs (such as selected nootropics) for Alzheimer disease** | No excluding services or diagnoses. | | | |
| **Opioids for acute non-specific back pain** | ***ICD*** |  |  |  |
|  | C00 – C97 | Malignant neoplasms | x | x |
|  | D37 – D48 | Neoplasms of uncertain or unknown behaviour | x | x |
|  | F11.2 | Mental and behavioural disorders due to use of opioids: Dependence syndrome | x | x |
|  | M00 – M25 | Arthropathies | x | x |
|  | M30 – M36 | Systemic connective tissue disorders | x | x |
|  | M40 – M43 | Deforming dorsopathies | x | x |
|  | M45 – M49 | Spondylopathies | x | x |
|  | M50 | Cervical disc disorders | x | x |
|  | M51 | Other intervertebral disc disorders | x | x |
|  | M53 | Other dorsopathies, not elsewhere classified | x | x |
|  | M60 – M79 | Soft tissue disorders | x | x |
|  | M80 – M94 | Osteopathies and chondropathies | x | x |
|  | M95 – M99 | Other disorders of the musculoskeletal system and connective tissue | x | x |
|  | Q35 – Q37 | Cleft lip and cleft palate | x | x |
|  | Q65 – Q79 | Congenital malformations and deformations of the musculoskeletal system | x | x |
|  | Q80 – Q89 | Congenital malformations and deformations of the musculoskeletal system | x | x |
|  | S00 – S09 | Injuries to the head | x | x |
|  | S10 – S19 | Injuries to the neck | x | x |
|  | S20 – S29 | Injuries to the thorax | x | x |
|  | S30 – S39 | Injuries to the abdomen, lower back, lumbar spine and pelvis | x | x |
|  | S40 – S49 | Injuries to the shoulder and upper arm | x | x |
|  | S50 – S59 | Injuries to the elbow and forearm | x | x |
|  | S60 – S69 | Injuries to the wrist and hand | x | x |
|  | S70 – S79 | Injuries to the hip and thigh | x | x |
|  | S80 – S89 | Injuries to the knee and lower leg | x | x |
|  | S90 – S99 | Injuries to the ankle and foot | x | x |
|  | T00 – T07 | Injuries involving multiple body regions | x | x |
|  | T08 – T14 | Injuries to unspecified part of trunk, limb or body region | x | x |
|  | T15 – T19 | Effects of foreign body entering through natural orifice | x | x |
|  | T20 – T32 | Burns and corrosions | x | x |
|  | T33 – T35 | Frostbite | x | x |
|  | T66 – T78 | Other and unspecified effects of external causes | x | x |
|  | T79 | Certain early complications of trauma, not elsewhere classified | x | x |
|  | T80 – T88 | Complications of surgical and medical care, not elsewhere classified | x | x |
|  | T89 | Other specified complications of trauma | x | x |
|  | T90 – T98 | Sequelae of injuries, of poisoning and o other consequences of external causes | x | x |
|  | V01 – X59 | Accidents | x | x |
|  | X60 – X84 | Intentional self-harm | x | x |
|  | X85 – Y09 | Assault | x | x |
|  | Y10 – Y34 | Event of undetermined intent | x | x |
|  | Y35 – Y36 | Legal intervention and operations of war | x | x |
|  | Y40 – Y84 | Complications of medical and surgical care | x | x |
| **Opioids for migraine or headache** | ***ICD*** |  |  |  |
|  | B20 – B24 | Human immunodeficiency virus [HIV] disease | x |  |
|  | C00 – C97 | Malignant neoplasms | x |  |
|  | D37 – D48 | Neoplasms of uncertain or unknown behaviour | x |  |
|  | F11.2 | Mental and behavioural disorders due to use of opioids: Dependence syndrome | x |  |
|  | G40 | Epilepsy | x |  |
|  | M00 – M99 | Diseases of the musculoskeletal system and connective tissue | x |  |
|  | Q35 – Q37 | Cleft lip and cleft palate | x |  |
|  | Q65 – Q79 | Congenital malformations and deformations of the musculoskeletal system | x |  |
|  | Q80 – Q89 | Congenital malformations and deformations of the musculoskeletal system | x |  |
|  | R10 | Abdominal and pelvic pain | x |  |
|  | R52.1 | Chronic intractable pain | x |  |
|  | S00 – S09 | Injuries to the head | x |  |
|  | S10 – S19 | Injuries to the neck | x |  |
|  | S20 – S29 | Injuries to the thorax | x |  |
|  | S30 – S39 | Injuries to the abdomen, lower back, lumbar spine and pelvis | x |  |
|  | S40 – S49 | Injuries to the shoulder and upper arm | x |  |
|  | S50 – S59 | Injuries to the elbow and forearm | x |  |
|  | S60 – S69 | Injuries to the wrist and hand | x |  |
|  | S70 – S79 | Injuries to the hip and thigh | x |  |
|  | S80 – S89 | Injuries to the knee and lower leg | x |  |
|  | S90 – S99 | Injuries to the ankle and foot | x |  |
|  | T00 – T07 | Injuries involving multiple body regions | x |  |
|  | T08 – T14 | Injuries to unspecified part of trunk, limb or body region | x |  |
|  | T15 – T19 | Effects of foreign body entering through natural orifice | x |  |
|  | T20 – T32 | Burns and corrosions | x |  |
|  | T33 – T35 | Frostbite | x |  |
|  | T66 – T78 | Other and unspecified effects of external causes | x |  |
|  | T79 | Certain early complications of trauma, not elsewhere classified | x |  |
|  | T80 – T88 | Complications of surgical and medical care, not elsewhere classified | x |  |
|  | T89 | Other specified complications of trauma | x |  |
|  | T90 – T98 | Sequelae of injuries, of poisoning and of other consequences of external causes | x |  |
|  | V01 – X59 | Accidents | x |  |
|  | X60 – X84 | Intentional self-harm | x |  |
|  | X85 – Y09 | Assault | x |  |
|  | Y10 – Y34 | Event of undetermined intent | x |  |
|  | Y35 – Y36 | Legal intervention and operations of war | x |  |
|  | Y40 – Y84 | Complications of medical and surgical care | x |  |
|  | Z32 | Pregnancy examination and test | x |  |
|  | Z33 | Pregnant state, incidental | x |  |
|  | Z34 | Supervision of normal pregnancy | x |  |
|  | Z35 | Supervision of high-risk pregnancy | x |  |
|  | Z64.0 | Problems related to unwanted pregnancy | x |  |
|  | ***OPS*** |  |  |  |
|  | 5-01 – 5-99 | Surgical procedures | x |  |
| **Diagnostic tests** | | | | |
| **Bone mineral density testing at frequent intervals** | ***ICD*** |  |  |  |
|  | S00 – S09 | Injuries to the head |  | x |
|  | S10 – S19 | Injuries to the neck |  | x |
|  | S20 – S29 | Injuries to the thorax |  | x |
|  | S30 – S39 | Injuries to the abdomen, lower back, lumbar spine and pelvis |  | x |
|  | S40 – S49 | Injuries to the shoulder and upper arm |  | x |
|  | S50 – S59 | Injuries to the elbow and forearm |  | x |
|  | S60 – S69 | Injuries to the wrist and hand |  | x |
|  | S70 – S79 | Injuries to the hip and thigh |  | x |
|  | S80 – S89 | Injuries to the knee and lower leg |  | x |
|  | S90 – S99 | Injuries to the ankle and foot |  | x |
|  | T00 – T07 | Injuries involving multiple body regions |  | x |
|  | T08 – T14 | Injuries to unspecified part of trunk, limb or body region |  | x |
|  | ***ATC*** |  |  |  |
|  | H02AB | Glucocorticoids |  | x |
|  | R03BA | Glucocorticoids |  | x |
| **Colonoscopy for constipation** | ***ICD*** |  |  |  |
|  | D50 – D53 | Nutritional anaemias | x | x |
|  | D55 – D59 | Haemolytic anaemias | x | x |
|  | D60 – D64 | Aplastic and other anaemias | x | x |
|  | D65 – D69 | Coagulation defects, purpura and other haemorrhagic conditions | x | x |
|  | R63.4 | Abnormal weight loss | x | x |
|  | Z80.0 | Family history of malignant neoplasm of digestive organs | x | x |
|  | Z85.0 | Personal history of malignant neoplasm of digestive organs | x | x |
|  | Z87.1 | Personal history of diseases of the digestive system | x | x |
| **EEG for headache** | ***ICD*** |  |  |  |
|  | F10.3 | Mental and behavioural disorders due to use of alcohol: Withdrawal state | x | x |
|  | F00 – F99 | Mental and behavioural disorders |  | x |
|  | G40 | Epilepsy | x | x |
|  | G43.3 | Complicated migraine | x | x |
|  | G00 – G99 | Diseases of the nervous system |  | x |
|  | R55 | Syncope and collapse | x | x |
|  | R56 | Convulsions, not elsewhere classified | x | x |
| **Endometrial biopsy for evaluation of infertility** | ***ICD*** |  |  |  |
|  | C00 – C97 | Malignant neoplasms | x | x |
| **Imaging for acute non-specific back pain** | ***ICD*** |  |  |  |
|  | C00 – C97 | Malignant neoplasms | x | x |
|  | D37 – D48 | Neoplasms of uncertain or unknown behaviour | x | x |
|  | M00 – M25 | Arthropathies | x | x |
|  | M30 – M36 | Systemic connective tissue disorders | x | x |
|  | M40 – M43 | Deforming dorsopathies | x | x |
|  | M45 – M49 | Spondylopathies | x | x |
|  | M50 | Cervical disc disorders | x | x |
|  | M51 | Other intervertebral disc disorders | x | x |
|  | M53 | Other dorsopathies, not elsewhere classified | x | x |
|  | M60 – M79 | Soft tissue disorders | x | x |
|  | M80 – M94 | Osteopathies and chondropathies | x | x |
|  | M95 – M99 | Other disorders of the musculoskeletal system and connective tissue | x | x |
|  | Q35 – Q37 | Cleft lip and cleft palate | x | x |
|  | Q65 – Q79 | Congenital malformations and deformations of the musculoskeletal system | x | x |
|  | Q80 – Q89 | Other concenital malformations | x | x |
|  | S00 – S09 | Injuries to the head | x | x |
|  | S10 – S19 | Injuries to the neck | x | x |
|  | S20 – S29 | Injuries to the thorax | x | x |
|  | S30 – S39 | Injuries to the abdomen, lower back, lumbar spine and pelvis | x | x |
|  | S40 – S49 | Injuries to the shoulder and upper arm | x | x |
|  | S50 – S59 | Injuries to the elbow and forearm | x | x |
|  | S60 – S69 | Injuries to the wrist and hand | x | x |
|  | S70 – S79 | Injuries to the hip and thigh | x | x |
|  | S80 – S89 | Injuries to the knee and lower leg | x | x |
|  | S90 – S99 | Injuries to the ankle and foot | x | x |
|  | T00 – T07 | Injuries involving multiple body regions | x | x |
|  | T08 – T14 | Injuries to unspecified part of trunk, limb or body region | x | x |
|  | T15 – T19 | Effects of foreign body entering through natural orifice | x | x |
|  | T20 – T32 | Burns and corrosions | x | x |
|  | T33 – T35 | Frostbite | x | x |
|  | T66 – T78 | Other and unspecified effects of external causes | x | x |
|  | T79 | Certain early complications of trauma, not elsewhere classified | x | x |
|  | T80 – T88 | Complications of surgical and medical care, not elsewhere classified | x | x |
|  | T89 | Other specified complications of trauma | x | x |
|  | T90 – T98 | Sequelae of injuries, of poisoning and of other consequences of external causes | x | x |
|  | V01 – X59 | Accidents | x | x |
|  | X60 – X84 | Intentional self-harm | x | x |
|  | X85 – Y09 | Assault | x | x |
|  | Y10 – Y34 | Event of undetermined intent | x | x |
|  | Y35 – Y36 | Legal intervention and operations of war | x | x |
|  | Y40 – Y84 | Complications of medical and surgical care | x | x |
| **Gastroscopy for dyspepsia** | ***ICD*** |  |  |  |
|  | D50 – D53 | Nutritional anaemias | x | x |
|  | D55 – D59 | Haemolytic anaemias | x | x |
|  | D60 – D64 | Aplastic and other anaemias | x | x |
|  | D65 – D69 | Coagulation defects, purpura and other haemorrhagic conditions | x | x |
|  | K21 | Gastro-oesophageal reflux disease |  | x |
|  | K22 | Perforation of oesophagus |  | x |
|  | K23 | Disorders of oesophagus in disease classified elsewhere |  | x |
|  | K25 | Gastric ulcer |  | x |
|  | K26 | Duodenal ulcer |  | x |
|  | K27 | Peptic ulcer, site unspecified |  | x |
|  | K28 | Gastrojejunal ulcer |  | x |
|  | K29 | Gastritis and duodenitis |  | x |
|  | K31 | Other diseases of stomach and duodenum |  | x |
|  | R13 | Dysphagia | x | x |
|  | R63.4 | Abnormal weight loss | x | x |
|  | Z80.0 | Family history of malignant neoplasm of digestive organs |  | x |
|  | Z85.0 | Personal history of malignant neoplasm of digestive organs |  | x |
|  | Z87.1 | Personal history of diseases of the digestive system |  | x |
| **Imaging for migraine or headache** | ***ICD*** |  |  |  |
|  | C69 – C72 | Malignant neoplasms of eye, brain and other parts of central nervous system | x | x |
|  | D80 - D89 | Certain disorders involving the immune mechanism | x | x |
|  | D90 | Immune compromise due to radiation, chemotherapy or other immunosuppressive measures | x | x |
|  | G40 | Epilepsy | x | x |
|  | G43.2 | Status migrainosus | x | x |
|  | G43.3 | Complicated migraine |  | x |
|  | G43.8 | Other migraine |  | x |
|  | G43.9 | Migraine, unspecified |  | x |
|  | G44.0 | Cluster headache syndrome | x | x |
|  | G44.1 | Vascular headache, not elsewhere classified | x | x |
|  | G44.3 | Chronic post-traumatic headache | x | x |
|  | G44.4 | Drug-induced headache, not elsewhere classified | x | x |
|  | G44.8 | Other specified headache syndromes | x | x |
|  | H47 | Other disorders of optic [2nd] nerve and visual pathways | x | x |
|  | R29.1 | Meningismus | x | x |
|  | R29.2 | Abnormal reflex | x | x |
|  | R29.3 | Abnormal posture | x | x |
|  | R29.5 | Neurological neglect syndrome | x | x |
|  | R29.6 | Tendency to fall, not elsewhere classified | x | x |
|  | R29.8 | Other and unspecified symptoms and signs involving the nervous and musculoskeletal systems | x | x |
|  | R50 | Fever of other and unknown origin | x | x |
|  | S02 | Fracture of skull and facial bones | x | x |
|  | S04 | Injury of cranial nerves | x | x |
|  | S06 | Intracranial injury | x | x |
|  | S07 | Crushing injury of head | x | x |
|  | S08 | Traumatic amputation of part of head | x | x |
|  | S09 | Other and unspecified injuries of head | x | x |
| **Preoperative chest radiography prior to selected surgeries** | ***ICD*** |  |  |  |
|  | C00 – C97 | Malignant neoplasms | x | x |
|  | E01 | Iodine-deficiency related thyroid disorders and allied conditions | x | x |
|  | E04 | Other nontoxic goitre | x | x |
|  | E05 | Thyrotoxicosis [hyperthyroidism] | x | x |
|  | I00 – I99 | Diseases of the circulatory system |  | x |
|  | J95 – J99 | Other diseases of the respiratory system |  | x |
|  | M40 – M43 | Deforming dorsopathies | x | x |
|  | ***EBM*** |  |  |  |
|  | 31181 | Cardiac surgery procedure with an incision-to-closure time up to 15 minutes | x | x |
|  | 31182 | Cardiac surgery procedure with an incision-to-closure time between 15 and 30 minutes | x | x |
|  | 31183 | Cardiac surgery procedure with an incision-to-closure time between 30 and 45 minutes | x | x |
|  | 31184 | Cardiac surgery procedure with an incision-to-closure time between 45 and 60 minutes | x | x |
|  | 31185 | Cardiac surgery procedure with an incision-to-closure time between 60 and 90 minutes | x | x |
|  | 31186 | Cardiac surgery procedure with an incision-to-closure time between 90 and 120 minutes | x | x |
|  | 31187 | Cardiac surgery procedure with an incision-to-closure time above 120 minutes | x | x |
|  | 31191 | Thoracic surgical procedure with an incision-to-closure time up to 15 minutes | x | x |
|  | 31192 | Thoracic surgical procedure with an incision-to-closure time between 15 and 30 minutes | x | x |
|  | 31193 | Thoracic surgical procedure with an incision-to-closure time between 30 and 45 minutes | x | x |
|  | 31194 | Thoracic surgical procedure with an incision-to-closure time between 45 and 60 minutes | x | x |
|  | 31195 | Thoracic surgical procedure with an incision-to-closure time between 60 and 90 minutes | x | x |
|  | 31196 | Thoracic surgical procedure with an incision-to-closure time between 90 and 120 minutes | x | x |
|  | 31197 | Thoracic surgical procedure with an incision-to-closure time above 120 minutes | x | x |
|  | ***OPS*** |  |  |  |
|  | 5-32 | Excision and resection in lung and bronchus | x | x |
|  | 5-33 | Other operations on lungs and bronchus | x | x |
|  | 5-34 | Operations on chest wall, pleura, mediastinum and diaphragm | x | x |
|  | 5-35 | Operations on cardiac valves and septa and vessels near the heart | x | x |
|  | 5-36 | Operations on the coronary vessels | x | x |
|  | 5-37 | Rhythm surgery and other operations on heart and pericardium | x | x |
| **Preoperative stress testing prior to selected surgeries** | ***ICD*** |  |  |  |
|  | I05 – I09 | Chronic rheumatic heart disease | x | x |
|  | I10 – I15 | Hypertensive diseases | x | x |
|  | I20 – I25 | Ischaemic heart diseases | x | x |
|  | I26 – I28 | Pulmonary heart disease and diseases of pulmonary circulation | x | x |
|  | I30 – I52 | Other forms of heart disease | x | x |
|  | R00 | Abnormalities of heart beat | x | x |
|  | ***EBM*** |  |  |  |
|  | 31181 | Cardiac surgery procedure with an incision-to-closure time up to 15 minutes | x | x |
|  | 31182 | Cardiac surgery procedure with an incision-to-closure time between 15 and 30 minutes | x | x |
|  | 31183 | Cardiac surgery procedure with an incision-to-closure time between 30 and 45 minutes | x | x |
|  | 31184 | Cardiac surgery procedure with an incision-to-closure time between 45 and 60 minutes | x | x |
|  | 31185 | Cardiac surgery procedure with an incision-to-closure time between 60 and 90 minutes | x | x |
|  | 31186 | Cardiac surgery procedure with an incision-to-closure time between 90 and 120 minutes | x | x |
|  | 31187 | Cardiac surgery procedure with an incision-to-closure time above 120 minutes | x | x |
|  | 31191 | Thoracic surgical procedure with an incision-to-closure time up to 15 minutes | x | x |
|  | 31192 | Thoracic surgical procedure with an incision-to-closure time between 15 and 30 minutes | x | x |
|  | 31193 | Thoracic surgical procedure with an incision-to-closure time between 30 and 45 minutes | x | x |
|  | 31194 | Thoracic surgical procedure with an incision-to-closure time between 45 and 60 minutes | x | x |
|  | 31195 | Thoracic surgical procedure with an incision-to-closure time between 60 and 90 minutes | x | x |
|  | 31196 | Thoracic surgical procedure with an incision-to-closure time between 90 and 120 minutes | x | x |
|  | 31197 | Thoracic surgical procedure with an incision-to-closure time above 120 minutes | x | x |
|  | ***OPS*** |  |  |  |
|  | 5-32 | Excision and resection in lung and bronchus | x | x |
|  | 5-33 | Other operations on lungs and bronchus | x | x |
|  | 5-34 | Operations on chest wall, pleura, mediastinum and diaphragm | x | x |
|  | 5-35 | Operations on cardiac valves and septa and vessels near the heart | x | x |
|  | 5-36 | Operations on the coronary vessels | x | x |
|  | 5-37 | Rhythm surgery and other operations on heart and pericardium | x | x |
| **Stress echocardiography for detection of coronary artery disease in ACS** | No excluding services or diagnoses. | | | |
| **Stress testing for stable coronary disease** | No excluding services or diagnoses. | | | |
| **Spirometry for known COPD** | No excluding services or diagnoses. | | | |
| **Testing for group A streptococcal pharyngitis** | ***ICD*** |  |  |  |
|  | Z20 | Contact with and exposure to communicable diseases |  | x |
| **Free T3/T4 level testing for hypothyroidism** | ***ICD*** |  |  |  |
|  | C75.1 | Malignant neoplasm: Pituitary gland |  | x |
|  | D09.3 | Carcinoma in situ: Thyroid and other endocrine glands |  | x |
|  | D17 | Benign lipomatous neoplasm |  | x |
|  | D44.3 | Neoplasm of uncertain or unknown behaviour: Pituitary gland |  | x |
|  | E00 | Congenital iodine-deficiency syndrome |  | x |
|  | E23 | Hypofunction and other disorders of pituitary gland |  | x |
|  | E24.0 | Pituitary-dependent Cushing disease |  | x |
|  | E85 | Amyloidosis |  | x |
|  | E89 | Postprocedural endocrine and metabolic disorders, not elsewhere classified |  | x |
|  | G04 | Encephalitis, myelitis and encephalomyelitis |  | x |
|  | M14.5 | Arthropathies in other endocrine, nutritional and metabolic disorders |  | x |
|  | S06 | Intracranial injury |  | x |
| **Tumour marker testing without cancer diagnosis** | ***ICD*** |  |  |  |
|  | B18.2 | Chronic viral hepatitis C |  | x |
|  | C00 – C97 | Malignant neoplasms | x | x |
|  | D00 – D09 | In situ neoplasms | x | x |
|  | K50 | Crohn disease [regional enteritis] |  | x |
|  | K51 | Ulcerative colitis |  | x |
|  | K74.3 | Primary biliary cirrhosis |  | x |
|  | K74.4 | Secondary biliary cirrhosis |  | x |
|  | K74.5 | Biliary cirrhosis, unspecified |  | x |
|  | K74.6 | Other and unspecified cirrhosis of liver |  | x |
|  | K86.1 | Other chronic pancreatitis |  | x |
|  | N83 | Noninflammatory disorders of ovary, fallopian tube and broad ligament |  | x |
|  | U55 | On waiting list for organ transplant |  | x |
|  | Z75.6 | Successful registration for organ transplantation without high urgency status |  | x |
|  | Z75.7 | Successful registration for organ transplantation with high urgency status |  | x |
|  | Z94 | Transplanted organ and tissue status |  | x |
| **Screening** | | | | |
| **Cancer screening for dialysis-dependent chronic kidney disease** | No excluding services or diagnoses. | | | |
| **Colorectal cancer screening for older persons** | ***ICD*** |  |  |  |
|  | C18 | Malignant neoplasm of colon |  | x |
|  | K63.5 | Polyp of colon |  | x |
|  | Z80.0 | Family history of malignant neoplasm of digestive organs |  | x |
| **Mammography screening for older women** | ***ICD*** |  |  |  |
|  | C50 | Malignant neoplasm of breast |  | x |
|  | N62 | Hypertrophy of breast |  | x |
|  | N63 | Unspecified lump in breast |  | x |
| **Mammography screening for younger women** | ***ICD*** |  |  |  |
|  | C50 | Malignant neoplasm of breast |  | x |
|  | N60 – N64 | Disorders of breast |  | x |
|  | Z80 | Family history of primary malignant neoplasm |  | x |
|  | ***EBM*** |  |  |  |
|  | 07345 | Supplementary lump sum for treatment and/or care of a patient with a confirmed oncological disease during ongoing oncological therapy or follow-up care |  | x |
|  | 08345 | Supplementary lump sum for treatment and/or care of a patient with a confirmed oncological disease during ongoing oncological therapy or follow-up care |  | x |
|  | ***ATC*** |  |  |  |
|  | G03C | Sex hormones and modulators of the genital system: Estrogens |  | x |
|  | G03D | Sex hormones and modulators of the genital system: Progestogens |  | x |
|  | G03F | Sex hormones and modulators of the genital system: Progestogens and estrogens in combination |  | x |
| **Treatment** | | | | |
| **Abdominal hysterectomy for benign diseases** | ***ICD*** |  |  |  |
|  | C00 – C97 | Malignant neoplasms | x | x |
|  | N73.6 | Female pelvic peritoneal adhesions |  | x |
|  | N80 | Endometriosis |  | x |
|  | N99.4 | Postprocedural pelvic peritoneal adhesions |  | x |
|  | O82 | Single delivery by caesarean section | x | x |
|  | Z80 | Family history of primary malignant neoplasm | x | x |
|  | ***OPS*** |  |  |  |
|  | 5-74 | Caesarean section and child development | x | x |
| **Chemotherapy for cancer in the last months of life** | No excluding services or diagnoses. | | | |
| **Electrotherapy for pressure ulcer** | ***ICD*** |  |  |  |
|  | L98.4 | Chronic ulcer of skin, not elsewhere classified |  | x |
| **ERC for acute gallstone pancreatitis without cholangitis** | ***ICD*** |  |  |  |
|  | K80.3 | Calculus of bile duct with cholangitis | x | x |
|  | K80.41 | Calculus of bile duct with cholecystitis with obstruction of biliary tract | x | x |
|  | K80.51 | Calculus of bile duct without cholangitis or cholecystitis with obstruction of biliary tract | x | x |
|  | K83.0 | Cholangitis | x | x |

| **Epidural steroid injections for low back pain** | ***ICD*** |  |  |  |
| --- | --- | --- | --- | --- |
|  | C00 – C97 | Malignant neoplasms | x | x |
|  | D37 – D48 | Neoplasms of uncertain or unknown behaviour | x | x |
|  | G55.1 | Nerve root and plexus compressions in intervertebral disc disorders | x | x |
|  | M00 – M25 | Arthropathies | x | x |
|  | M30 – M36 | Systemic connective tissue disorders | x | x |
|  | M40 – M43 | Dorsopathies | x | x |
|  | M45 – M49 | Spondylopathies | x | x |
|  | M50 | Cervical disc disorders | x | x |
|  | M51 | Other intervertebral disc disorders | x | x |
|  | M53 | Other dorsopathies, not elsewhere classified | x | x |
|  | M54.15 | Radiculopathy thoracolumbar region | x | x |
|  | M54.16 | Radiculopathy lumbar region | x | x |
|  | M54.17 | Radiculopathy lumbosacral region | x | x |
|  | M54.4 | Lumbago with sciatica | x | x |
|  | M60 – M79 | Soft tissue disorders | x | x |
|  | M80 – M94 | Osteopathies and chondropathies | x | x |
|  | M95 – M99 | Other disorders of the musculoskeletal system and connective tissue | x | x |
|  | Q35 – Q37 | Cleft lip and cleft palate | x | x |
|  | Q65 – Q79 | Congenital malformations and deformations of the musculoskeletal system | x | x |
|  | Q80 – Q89 | Other congenital malformations | x | x |
|  | S00 – S09 | Injuries to the head | x | x |
|  | S10 – S19 | Injuries to the neck | x | x |
|  | S20 – S29 | Injuries to the thorax | x | x |
|  | S30 – S39 | Injuries to the abdomen, lower back, lumbar spine and pelvis | x | x |
|  | S40 – S49 | Injuries to the shoulder and upper arm | x | x |
|  | S50 – S59 | Injuries to the elbow and forearm | x | x |
|  | S60 – S69 | Injuries to the wrist and hand | x | x |
|  | S70 – S79 | Injuries to the hip and thigh | x | x |
|  | S80 – S89 | Injuries to the knee and lower leg | x | x |
|  | S90 – S99 | Injuries to the ankle and foot | x | x |
|  | T00 – T07 | Injuries involving multiple body regions | x | x |
|  | T08 – T14 | Injuries to unspecified part of trunk, limb or body region | x | x |
|  | T15 – T19 | Effects of foreign body entering through natural orifice | x | x |
|  | T20 – T32 | Burns and corrosions | x | x |
|  | T33 – T35 | Frostbite | x | x |
|  | T66 – T78 | Other and unspecified effects of external causes | x | x |
|  | T79 | Certain early complications of trauma, not elsewhere classified | x | x |
|  | T80 – T88 | Complications of surgical and medical care, not elsewhere classified | x | x |
|  | T89 | Other specified complications of trauma | x | x |
|  | T90 – T98 | Sequelae of injuries, of poisoning and of other consequences of external causes | x | x |
|  | V01 – X59 | Accidents | x | x |
|  | X60 – X84 | Intentional self-harm | x | x |
|  | X85 – Y09 | Assault | x | x |
|  | Y10 – Y34 | Event of undetermined intent | x | x |
|  | Y35 – Y36 | Legal intervention and operations of war | x | x |
|  | Y40 – Y84 | Complications of medical and surgical care | x | x |
| **Inhalation therapy for COPD without previously confirming the diagnosis by spirometry** | ***EBM*** |  |  |  |
|  | 03330 | Spirographic examination | x | x |
|  | 27330 | Spirographic examination | x | x |
|  | 13255 | Spirographic examination | x | x |
| **PTA of the renal artery or stenting for selected diagnoses** | ***ICD*** |  |  |  |
|  | I77.3 | Arterial fibromuscular dysplasia | x | x |
|  | J81 | Pulmonary oedema | x | x |
| **Postoperative radiation therapy after radical prostatectomy** | No excluding services or diagnoses. | | | |
| **Removal of gallbladder during bariatric surgery** | ***ICD*** |  |  |  |
|  | C23 | Malignant neoplasm of gallbladder | x |  |
|  | D01.5 | Carcinoma in situ: Liver, gallbladder and bile ducts | x |  |
|  | D37.6 | Neoplasm of uncertain or unknown behaviour: Liver, gallbladder and bile ducts | x |  |
|  | K80.00 | Calculus of gallbladder with acute cholecystitis: Without mention of obstruction of biliary tract | x |  |
|  | K80.01 | Calculus of gallbladder with acute cholecystitis: With obstruction of biliary tract | x |  |
|  | K80.10 | Calculus of gallbladder with other cholecystitis: Without mention of obstruction of biliary tract | x |  |
|  | K80.11 | Calculus of gallbladder with other cholecystitis: With obstruction of biliary tract | x |  |
|  | K80.20 | Calculus of gallbladder without cholecystitis: Without mention of obstruction of biliary tract | x |  |
|  | K80.21 | Calculus of gallbladder without cholecystitis: With obstruction of biliary tract | x |  |
|  | K82 | Other diseases of gallbladder | x |  |
|  | K87 | Disorders of gallbladder, biliary tract and pancreas in diseases classified elsewhere | x |  |
|  | Q44.0 | Agenesis, aplasia and hypoplasia of gallbladder | x |  |
|  | Q44.1 | Other congenital malformations of gallbladder | x |  |
| **Retinal laser therapy or cryotherapy for asymptomatic lattice degeneration** | ***ICD*** |  |  |  |
|  | H33 | Retinal detachments and breaks | x | x |

| **Spinal fusion for low back pain** | ***ICD*** |  |  |  |
| --- | --- | --- | --- | --- |
|  | G55.1 | Nerve root and plexus compressions in intervertebral disc disorders | x | x |
|  | M40.00 | Postural kyphosis: Multiple sites in spine | x | x |
|  | M40.06 | Postural kyphosis: Lumbar region | x | x |
|  | M40.07 | Postural kyphosis: Lumbosacral region | x | x |
|  | M40.08 | Postural kyphosis: Sacral and sacrococcygeal region | x | x |
|  | M40.09 | Postural kyphosis: Site unspecified | x | x |
|  | M40.10 | Other secondary kyphosis: Multiple sites in spine | x | x |
|  | M40.16 | Other secondary kyphosis: Lumbar region | x | x |
|  | M40.17 | Other secondary kyphosis: Lumbosacral region | x | x |
|  | M40.18 | Other secondary kyphosis: Sacral and sacrococcygeal region | x | x |
|  | M40.19 | Other secondary kyphosis: Site unspecified | x | x |
|  | M40.30 | Flatback syndrome: Multiple sites in spine | x | x |
|  | M40.36 | Flatback syndrome: Lumbar region | x | x |
|  | M40.37 | Flatback syndrome: Lumbosacral region | x | x |
|  | M40.38 | Flatback syndrome: Sacral and sacrococcygeal region | x | x |
|  | M40.39 | Flatback syndrome: Site unspecified | x | x |
|  | M40.40 | Other lordosis: Multiple sites in spine | x | x |
|  | M40.46 | Other lordosis: Lumbar region | x | x |
|  | M40.47 | Other lordosis: Lumbosacral region | x | x |
|  | M40.48 | Other lordosis: Sacral and sacrococcygeal region | x | x |
|  | M40.49 | Other lordosis: Multiple sites in spine | x | x |
|  | M41 | Scoliosis | x | x |
|  | M42 | Spinal osteochondrosis | x | x |
|  | M43.1 | Spondylolisthesis | x | x |
|  | M43.20 | Other fusion of spine: Multiple sites in spine | x | x |
|  | M43.26 | Other fusion of spine: Lumbar region | x | x |
|  | M43.27 | Other fusion of spine: Lumbosacral region | x | x |
|  | M43.28 | Other fusion of spine: Sacral and sacrococcygeal region | x | x |
|  | M43.29 | Other fusion of spine: Site unspecified | x | x |
|  | M43.3 | Recurrent atlantoaxial subluxation with myelopathy | x | x |
|  | M43.4 | Other recurrent atlantoaxial subluxation | x | x |
|  | M43.50 | Other recurrent vertebral subluxation: Multiple sites in spine | x | x |
|  | M43.56 | Other recurrent vertebral subluxation: Lumbar region | x | x |
|  | M43.57 | Other recurrent vertebral subluxation: Lumbosacral region | x | x |
|  | M43.58 | Other recurrent vertebral subluxation: Sacral and sacrococcygeal region | x | x |
|  | M43.59 | Other recurrent vertebral subluxation: Site unspecified | x | x |
|  | M43.6 | Torticollis | x | x |
|  | M43.80 | Other specified deforming dorsopathies: Multiple sites in spine | x | x |
|  | M43.86 | Other specified deforming dorsopathies: Lumbar region | x | x |
|  | M43.87 | Other specified deforming dorsopathies: Lumbosacral region | x | x |
|  | M43.88 | Other specified deforming dorsopathies: Sacral and sacrococcygeal region | x | x |
|  | M43.89 | Other specified deforming dorsopathies: Site unspecified | x | x |
|  | M43.90 | Deforming dorsopathy, unspecified: Multiple sites in spine | x | x |
|  | M43.96 | Deforming dorsopathy, unspecified: Lumbar region | x | x |
|  | M43.97 | Deforming dorsopathy, unspecified: Lumbosacral region | x | x |
|  | M43.98 | Deforming dorsopathy, unspecified: Sacral and sacrococcygeal region | x | x |
|  | M43.99 | Deforming dorsopathy, unspecified: Site unspecified | x | x |
|  | M51.1 | Lumbar and other intervertebral disc disorders with radiculopathy | x | x |
|  | M54.4 | Lumbago with sciatica | x | x |
|  | M79.65 | Pain in limb: Pelvic region and thigh | x | x |
|  | M79.66 | Pain in limb: Lower leg | x | x |
|  | M79.67 | Pain in limb: Ankle and foot | x | x |
|  | Q67.5 | Congenital deformity of spine | x | x |
|  | Q76.21 | Congenital spondylolisthesis | x | x |
|  | Q76.3 | Congenital scoliosis due to congenital bony malformation | x | x |
|  | Q76.4 | Other congenital malformations of spine, not associated with scoliosis | x | x |
| **Surgery for vesicoureteral reflux** | No excluding services or diagnoses. | | | |
| **Tube feeding via PEG in the last months of life** | No excluding services or diagnoses. | | | |
| **Unblocking nasolacrimal duct** | No excluding services or diagnoses. | | | |
