# Supplementary figures and images for: Selecting indicators for the measurement of low-value care using German claims data: A three-round modified Delphi panel
Source: PLoS One. 2025 Feb 18;20(2):e0314864. doi: 10.1371/journal.pone.0314864 (PMC11835324; doi:10.1371/journal.pone.0314864)

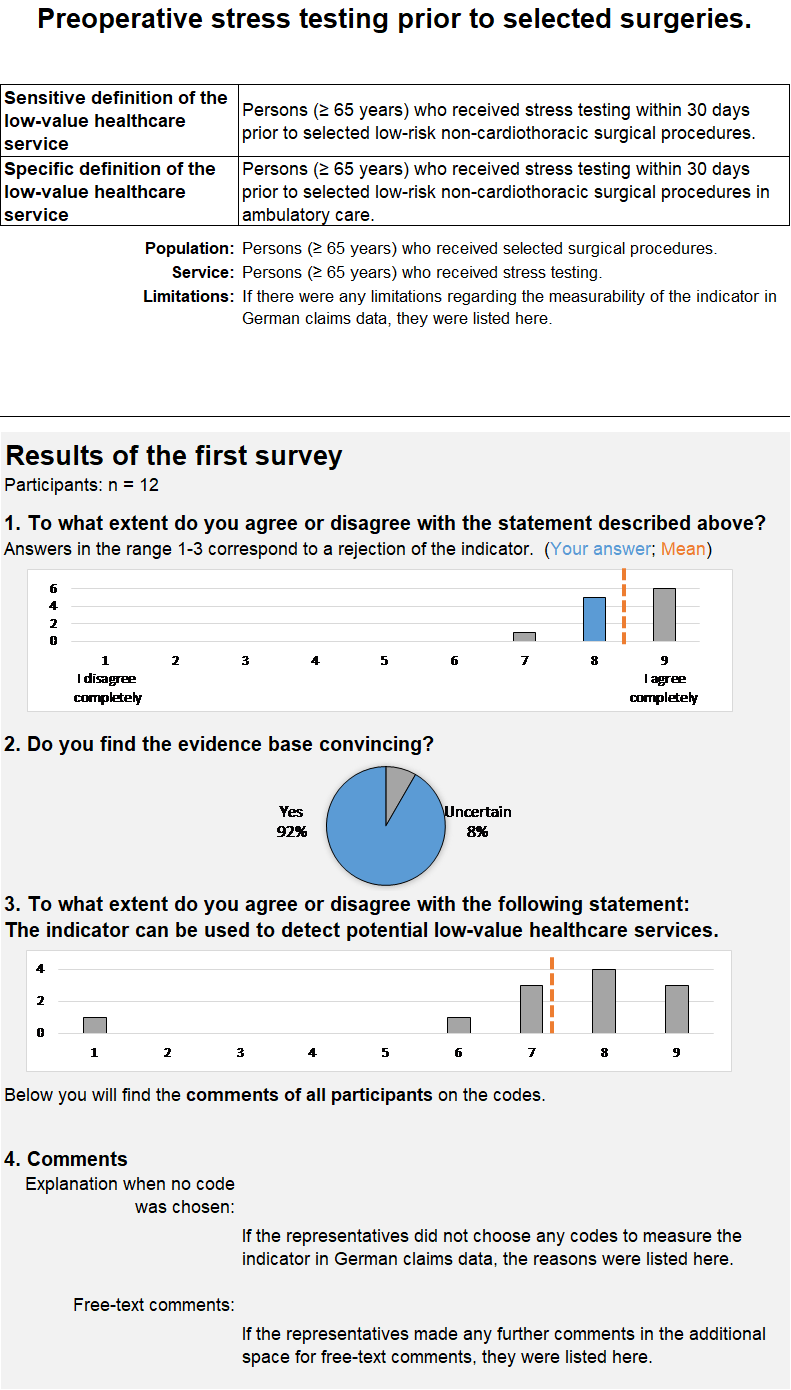

Supplement: S1 Fig — (TIF) [file pone.0314864.s005.tif]
