# Supplementary material for: Selecting indicators for the measurement of low-value care using German claims data: A three-round modified Delphi panel
Source: PLoS One. 2025 Feb 18;20(2):e0314864. doi: 10.1371/journal.pone.0314864 (PMC11835324; doi:10.1371/journal.pone.0314864)
Supplement: S1 File — (DOCX) [file pone.0314864.s006.docx]

# **S3: Evidence example of one indicator**

## Indicator: Free T3/T4 level testing for hypothyroidism

**1. The indicator is based on the following source**

- Schwartz AL, Jena AB, Zaslavsky AM, McWilliams JM. Analysis of Physician Variation in Provision of Low-Value Services. JAMA Intern Med. 2019;179:16–25. doi:10.1001/jamainternmed.2018.5086.

**2. The indicator can be associated with the following recommendations**

- Don’t use Free T4 or T3 to screen for hypothyroidism or to monitor and adjust levothyroxine (T4) dose in patients with known primary hypothyroidism, unless the patient has suspected or known pituitary or hypothalamic disease. Choosing Wisely® Canada. Canadian Society of Endocrinology and Metabolism (updated November 2020).

*Rationale:*

*T4 is converted into T3 at the cellular level in virtually all organs. Intracellular T3 levels regulate pituitary secretion and blood levels of TSH, as well as the effects of thyroid hormone in multiple organs. Therefore, in most people a normal TSH indicates either normal endogenous thyroid function or an adequate T4 replacement dose. TSH only becomes unreliable in patients with suspected or known pituitary or hypothalamic disease when TSH cannot respond physiologically to altered levels of T4 or T3. Patients should have access to additional testing, as required.*

- Don’t order a total or free T3 level when assessing levothyroxine (T4) dose in hypothyroid patients. Choosing Wisely® USA. Endocrine Society (updated July 2017).

*Rationale:*

*T4 is converted into T3 at the cellular level in virtually all organs. Intracellular T3 levels regulate pituitary secretion and blood levels of TSH, as well as the effects of thyroid hormone in multiple organs. However, T3 levels in blood are not reliable indicators of intracellular T3 concentration. Compared to patients with intact thyroid glands, patients taking T4 may have higher blood T4 and lower blood T3 levels. There is controversy as to whether a normal TSH reflects adequate intracellular T3 levels in all organs, However, even in patients taking both levothyroxine and liothyronine, there are no data suggesting that the blood level of total or free T3 correlates with a patient’s clinical response. Therefore, in most patients a normal TSH indicates a correct dose of T4.*

- [Don’t order a total or free T3 level when assessing thyroxine dose in hypothyroid patients. Choosing Wisely® Australia. Endocrine Society of Australia (released November 2015)](https://www.choosingwisely.org.au/recommendations/esa5).

*Rationale:*

*T4 (thyroxine) is converted into T3 at the cellular level in virtually all organs. Intracellular T3 levels regulate pituitary secretion and blood levels of thyroid-stimulating hormone (TSH), as well as the effects of thyroid hormone in multiple organs; a normal TSH indicates an adequate T4 dose. Conversion of T4 to T3 at the cellular level may not be reflected in the T3 level in the blood. Compared to patients with intact thyroid glands, patients with hypothyroidism taking T4 may have higher blood T4 and lower blood T3 levels. Thus the blood level of total or free T3 may be misleading (low normal or slightly low); in most patients a normal TSH indicates a correct dose of T4.*

**3. Level of Evidence**

- **Sources of the indicator**: Schwartz AL, Jena AB, Zaslavsky AM, McWilliams JM. Analysis of Physician Variation in Provision of Low-Value Services. JAMA Intern Med. 2019;179:16–25. doi:10.1001/jamainternmed.2018.5086

**Source refers to:** Choosing Wisely® USA

- **Sources of the indicator:** Choosing Wisely® Canada, USA and Australia

**Sources refer to:** Garber JR, Cobin RH, Gharib H, et al. Clinical practice guidelines for hypothyroidism in adults: cosponsored by the American Association of Clinical Endocrinologists and the American Thyroid Association [published correction appears in Endocr Pract. 2013;19:175]. Endocr Pract. 2012;18:988-1028. doi:10.4158/EP12280.GL

**Total level of evidence according to Oxford Level of Evidence: Level of Evidence 4/5**

Evidence-based guideline 2012 (without evidence of search strategies and evidence selection/evaluation): TSH should be used for screening: Measurement T3 in special cases. TSH should also be used to adjust medication of L-thyroxine. Cited some observational studies with observation that TSH is most sensitive parameter for change. Rapid Review CADTH 2015: Three observational studies, only one examined T3. Studies are inconclusive regarding benefit or lack of benefit of T3.

**4. German evidence-based guidelines:**

- Krude H. Diagnostik, Therapie und Verlaufskontrolle der primären angeborenen Hypothyreose, Klasse S2k. [Diagnostics, therapy and follow-up of primary congenital hypothyroidism]. AWMF-Register Nr. 027/017. 2011. <http://www.awmf.org>

Cave: only for primary congenital hypothyroidism.
