# Supplementary material for: Selecting indicators for the measurement of low-value care using German claims data: A three-round modified Delphi panel
Source: PLoS One. 2025 Feb 18;20(2):e0314864. doi: 10.1371/journal.pone.0314864 (PMC11835324; doi:10.1371/journal.pone.0314864)
Supplement: S2 File — (DOCX) [file pone.0314864.s007.docx]

# **S5: Medical specialties in the Delphi Panel**

The representatives represented 32 separate medical specialties, namely: eleven internal medicine sub-specialties (angiology, cardiology, endocrinology, gastroenterology, general internal medicine, geriatrics, haematology, oncology, infectious disease, nephrology, pneumology), eleven surgical specialties (general surgery, gynaecology, neurosurgery, orthopaedic surgery, otorhinolaryngology, paediatric surgery, plastic surgery, trauma surgery, urology, vascular surgery, visceral surgery), two paediatric sub-specialties (paediatric gastroenterology, paediatric infectious disease), anaesthesiology, family medicine, neurology, ophthalmology, physical medicine and rehabilitation, psychiatry and psychotherapy, radiology, and radiotherapy.
